# Bacterially sensitive nanoparticle-based dissolving microneedles of doxycycline for enhanced treatment of bacterial biofilm skin infection: A proof of concept study

**DOI:** 10.1016/j.ijpx.2020.100047

**Published:** 2020-04-14

**Authors:** Andi Dian Permana, Maria Mir, Emilia Utomo, Ryan F. Donnelly

**Affiliations:** a School of Pharmacy, Queen's University Belfast, Medical Biology Centre, 97 Lisburn Road, Belfast BT9 7BL, United Kingdom; b Department of Pharmaceutics, Faculty of Pharmacy, Hasanuddin University, Makassar, Indonesia; c Department of Pharmacy, Faculty of Biological Sciences, Quaid-i-Azam University, Islamabad 45320, Pakistan

**Keywords:** Doxycycline, Nanoparticles, Biofilm, Microneedles, *Staphylococcus aureus*, *Pseudomonas aeruginosa*

## Abstract

The presence of bacterial biofilms in wounds is a main issue in the healing process. Conventional therapy of bacterial biofilms is hampered by the poor penetration of antibacterial agents through the physical barrier on the infected skin and the non-specific target of antibacterial agents. Here, we present a combination approach of bacterial sensitive nanoparticles (NPs) and dissolving microneedles (MNs) of doxycycline (DOX) for improved biofilm penetration and specifically delivering DOX to the infection site. The NPs were prepared from poly(lactic-*co*-glycolic acid) and poly (Ɛ-caprolactone) decorated with chitosan. The release of DOX was improved with the presence of bacterial producing biofilm up to 7-fold. The incorporation of these NPs into dissolving MNs was able to significantly enhance the dermatokinetic profiles of DOX, indicated by higher retention time compared to needle-free patches. Importantly, the antibiofilm activity in *ex vivo* biofilm model showed that after 48 h, the bacterial bioburdens decreased up to 99.99% following the application of this approach. The results presented here assist as proof of principle for the improvement of dermatokinetic profiles and antibiofilm activities of DOX, following its formulation into bacterial sensitive NPs and delivery *via* MN. Future studies must explore *in vivo* efficacy in a suitable animal model.

## Graphical abstract

**Figure:**
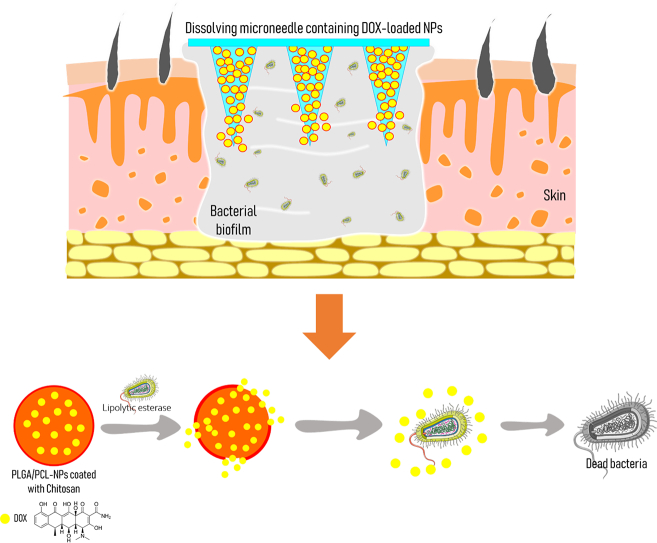

## 1 Introduction

Burn and chronic wounds are difficult to heal and need extended management thanks to several clinical complications (Alhusein et al., 2016; Hammond et al., 2011; Moore et al., 2006). Additionally, they significantly affect the life quality of patients and are one of the main health concerns for care systems globally. Importantly, it has been reported that approximately $50 billion are consumed *per annum* on care management of chronic wounds (El-Mohri et al., 2017). In addition to surgical debridement (including necrotic and infected tissue removal), the systemic and/or topical administration of antimicrobial agents for an extended period as the most recent therapeutic strategy for burn and chronic wounds could result in undesired systemic side effects (Haidar et al., 2020; Mihai et al., 2018). Furthermore, over approximately 80% of chronic wounds are assumed to be related with biofilm formation of bacteria (Duckworth et al., 2018). Despite the fact that several bacterial pathogens are involved, *Staphylococcus aureus* (SA) and *Pseudomonas aeruginosa* (PA) are the most commonly-isolated pathogens (Clinmicrorevs et al., 2001; Doern et al., 1999).

Bacterial biofilms are defined as a multifarious bacterial community attached as aggregates and surrounded within polysaccharides, adhesive pili, extracellular DNA, lipids and protein, referred to as a self-synthesized matrix of hydrated extracellular polymeric substances (EPS) (Flemming and Wingender, 2010; Olsen, 2015). In these cases, due to the fact that planktonic bacteria encapsulated in a biofilm matrix can display up to 1000-times more antibiotic resistance, conventional antibiotic treatments are often unsuccessful (Olsen, 2015). Unsurprisingly, since the currently used antibiotics have been primarily used to target planktonic bacteria, they are, therefore, unsuccessful in eliminating biofilm bacteria (Parsons and Summers, 2017). Importantly, biofilms have been recognized as a key impediment to the process of wound healing (Duckworth et al., 2018). Current biofilm removal process utilizes bleach or other erosive compounds to sterilize the wound (Marion-Ferey et al., 2003), resulting in low patient compliance and high health care expenses(Lynch and Robertson, 2008). Removal debridement by surgery of infected wounds can completely eliminate biofilm of the wound area. Nevertheless, it has been reported that biofilms are observed again after 2 days of the first removal (Wolcott et al., 2010), and the long-term antibiotic treatment further increases the appearance of antibiotic-resistant bacteria (Levy and Bonnie, 2004). Accordingly, the development of a novel drug delivery system as therapeutic chronic wound management which can disrupt and kill the bacterial biofilm is urgently warranted.

Several antimicrobial agents have been widely used in burn and chronic wounds. Doxycycline (DOX) is one of the agents which can effectively kill SA and PA (Adhirajan et al., 2009; Sader et al., 2002). The utilization of biodegradable nanoparticles (NPs) has been considered as a promising antimicrobial approach for efficient therapeutic management of infection (Guo et al., 2016). In order to improve the effectiveness of therapeutic agent, the NPs formulation has been modified by enhancing the targeting efficiency or responding to adjacent inducements, *i.e.* enzyme, pH or temperature (Allen and Cullis, 2004). With respect to the selection of biodegradable polymers, it has been reported that lipolytic esterase produced by SA and PA were able to initiate the biocatalytic hydrolysis of poly(lactic-*co*-glycolic acid) (PLGA) in ultrafine fiber formulations (Said et al., 2012, Said et al., 2011) and poly (Ɛ-caprolactone) (PCL) in nanogel formulations (Xiong et al., 2012). Therefore, these polymers can be considered as suitable polymers to selectively deliver antimicrobial agents to the infected site only. As a result, this delivery could potentially reduce the exposure to undesired sites, leading to a safe treatment strategy. Lately, the decoration of NPs charge with specific ligands has been conducted to modify the delivery of NPs. Amongst several types of ligands, chitosan is one of the generally used in NP formulations (Badran et al., 2018). In terms of bacterial biofilm targeting, chitosan has been reported to be biocompatible and biodegradable, and to possess an antimicrobial activity (Rabea et al., 2003). Chitosan NPs have been successfully utilized against 24-h-old *P. aeruginosa* biofilms of six strains isolated from clinical studies (Machul et al., 2015). Chitosan NPs possess a positive surface charge, whereas the surfaces of biofilm EPS and bacterial cell walls are negative. Accordingly, chitosan is anticipated to exhibit a high attraction to infected areas (Han et al., 2017). Leading on from these, the incorporation of chitosan onto either a PLGA or a PCL surface could potentially increase the effectiveness of biofilm targeting.

To penetrate the dense physical obstacle that biofilms present, the choice of a suitable dosage form is crucial. The systemic administration of antimicrobial agents is not able to deliver the drugs successfully to the desired infection site (Noel et al., 2010). Interestingly, in burn and chronic wounds, in addition to biofilm, the presence of necrotic tissue overlying the wound bed is another challenge in treatment (Caffarel-Salvador et al., 2015). Conventional topical dosage forms, *i.e.* creams, dressings and gels, have been reported to exhibit poor penetration of drug to this barrier, resulting in low drug concentration in the infected site (Bharambe et al., 2013; Lipsky and Hoey, 2009). Despite the fact that the nanocarriers have been broadly investigated for their efficacy in antimicrobial drug delivery, only a few investigations exist focusing on the delivery of nanoparticles to the biofilm. With this in mind, a new drug device which can increase the penetrability of antimicrobials across the necrotic tissue is required. This would avoid the necessity of necrotic tissue removal before the application of the conventional dosage forms, mentioned previously, in clinics. Dissolving microneedles (MNs) are a drug device which can by-pass the major skin barrier (Permana et al., 2019a). Essentially, MNs can provide a painless, localized, rapid delivery and patient-compliant administration approach. Unlike hypodermic needle injections, dissolving MNs are self-dissolvable and are prepared from biocompatible and biodegradable polymers. Thus, their utilization does not produce any biohazardous sharps waste (Permana et al., 2019c). Considering the advantages of this technology, the incorporation of NPs into dissolving MNs could potentially improve the amount of NPS penetrating the necrotic tissue of infected skin, and, as such, could potentially increase the effectiveness of burns and chronic wound management.

In this study, we present, for the first time, the development of NPs loaded with DOX incorporated into dissolving MNs as an innovative approach for potential improved treatment of chronic wounds with bacterial biofilms. PLGA and PCL were utilized as the polymer matrixes of the NPs, coated with chitosan to produce positively charged NPs. The NPs were characterized for their size, polydispersity index, zeta potential, shape, antibacterial activities and antibiofilm activities. Specifically, the release behavior of DOX in NPs was carried out with and without the presence of bacteria commonly associated with chronic wounds, namely SA and PA. Following this, the NPs were further loaded into dissolving MNs and the MNs were assessed for mechanical and insertion properties. Furthermore, to evaluate the effectiveness this approach, *ex vivo* dermatokinetic studies were carried out in normal porcine skin and *ex vivo* neonatal porcine skin biofilm model. Finally, the ability to penetrate and kill the bacterial biofilms were performed in *ex vivo* skin biofilm model. This is the first study investigating the effectiveness of the combination approach of NPs and MNs in *ex vivo* porcine skin biofilm model. The results of these proof of concept studies point towards the potential utilization of this combination delivery system to overcome the challenge in the treatment of bacterial biofilm infections in the skin.

## 2 Materials and methods

### 2.1 Materials

Doxycycline hyclate (DOX) (purity, ≥98%) of analytical grade was purchased from Alfa Aesar (Lancashire, UK). Acetic acid, chitosan (low-molecular-weight: 50–190 kDa), dichloromethane (DCM), poly(vinyl alcohol) (PVA) (31–50 kDa), PVA (9–10 kDa), poly (Ɛ-caprolactone) (PCL) (45 kDa) and sodium tripolyphosphate (TPP) were purchased from Sigma-Aldrich (Dorset, UK). Poly(lactic-*co*-glycolic acid) 40–75 kDa) was purchased from Lakeshore Biomaterials (Birmingham, AL). Poly(vinylpyrrolidone) PVP (58 kDa) was provided by Ashland (Kidderminster, UK). Ultrapure water was obtained from a water purification system (Elga PURELAB DV 25, Veolia Water Systems, Dublin, Ireland). All other reagents were of analytical grade and purchased from standard commercial suppliers.

### 2.2 Formulation of DOX-loaded NPs

A double emulsion (water-in-oil-in-water) (W/O/W) solvent evaporation method was applied to prepare DOX loaded PLGA (NP-1) and PCL NPs (NP-2) (Badran et al., 2018). Table 1 shows the composition of the NPs. In brief, DOX solution in water (1 mL) was emulsified in PLGA or PCL solution in dichloromethane (DCM) (2 mL) in a probe sonicator (at an amplitude of 80% with 10 s pulse on and 5 s pulse off) for 1 min, producing a water-in-oil (W/O) emulsion. Afterwards, this emulsion was emulsified in 5 mL PVA (9–10 kDa) solution in water (2% w/v) in a probe sonicator (at an amplitude of 80% with 10 s pulse on and 5 s pulse off) for 5 min, forming a W/O/W emulsion. To remove the DCM, this double emulsion was stirred for 6 h at room temperature. Finally, the formed NPs were collected after three washing cycles with distilled water by centrifugation at 14,000 rpm (Sigma® 1–14 micro-centrifuge, SciQuip Ltd., Shropshire, UK) for 30 min to remove the free DOX and the PVA solution.

**Table 1:** Composition of formulations NPs laded with DOX.

Composition	NP-1	NP-2	NP-3	NP-4	NP-5
PLGA (mg/2 mL)	50	–	50	–	–
PCL (mg/2 mL)	–	50	–	50	–
Chitosan (mg/5 mL)	–	–	50	50	50
TPP (mg/2.5 mL)	–	–	–	–	25
DOX (mg/mL)	25	25	25	25	25

To prepare PLGA (NP-3) and PCL (NP-4) NPs-coated with chitosan, NPs pellets collected after washing were dispersed in 5 mL distilled water. These dispersions were further mixed with the same volume of chitosan solutions (in 0.5% v/v acetic acid). The mixtures were then stirred for 2 h to allow the formation of NPs-coated chitosan. The formed NPs-coated chitosan was collected using the same procedure as uncoated NPs.

The chitosan NPs (NP-5) were prepared by an ionotropic gelation technique (Mili et al., 2018), with slight modifications. DOX and tripolyphosphate (TPP) were dissolved in distilled water. This solution was gently added to chitosan solution in 0.5% v/v acetic acid (in water) while stirred at 1000 rpm at room temperature post addition for 1 h. The formed NPs were collected using the same protocols as the PLGA and PCL NPs.

### 2.3 Characterization of DOX-loaded NPs

The particle sizes, polydispersity indices (PDI) and zeta potentials were determined using a NanoBrook Omni particle sizer and zeta potential analyzer (Brookhaven, New York, USA).

The encapsulations efficiency (EE) of DOX in NPs formulations were quantified by an indirect method (Badran et al., 2018). The amount of free DOX in the supernatant, following three washing cycles, was quantified using the HPLC method described in the analytical section. Finally, the EE of DOX was calculated using Eq. (1).

(1) $$\mathrm{EE}\;\left(\%\right)=\frac{{\mathrm{DOX}}_{\mathrm{total}}-{\mathrm{DOX}}_{\mathrm{free}}}{{\mathrm{DOX}}_{\mathrm{total}}}\times 100\%$$

To determine the drug loading (DL%) of DOX in NPs formulations, NPs dispersions were freeze dried (Virtis Advantage Bench-top Freeze-drier system, SP Scientific, Warminster, PA, USA) for 24 h, forming dry NPs. For PLGA NPs, PCL NPs as well as PLGA and PCL NPs-coated with chitosan, 10 mg of freeze-dried NPs was firstly dispersed in 5 mL of distilled water. Acetone (2 mL) was added to the dispersion to disrupt the NPs and the mixture was sonicated in a bath sonicator for 1 h. For chitosan NPs, 10 mg of freeze-dried NPs was dissolved in 10 mL of acetic acid solution (0.5% *v*/v) and was sonicated in a bath sonicator for 1 h. The suspension was then centrifuged at 14,000 rpm for 15 min and the amount of DOX in supernatant was quantified by HPLC. The DL was calculated using Eq. (2).

(2) $$\mathrm{DL}\;\left(\%\right)=\frac{\mathrm{Amount of encapsulated}\;\mathrm{DOX}}{\mathrm{Total weight}}\times 100\%$$

The morphologies of the DOX-loaded NPs were investigated using a scanning electron microscope (SEM) TM3030 (Hitachi, Krefeld, Germany). The interactions between each component in the formulations were studied using a Fourier transform infrared (FTIR) spectrometer (Accutrac FT/IR-4100™ Series, Jasco, Essex, UK). Differential scanning calorimetry (DSC) analysis of DOX, polymers, physical mixtures and DOX-loaded NPs were performed using a differential scanning calorimeter (DSC 2920, TA Instruments, Surrey, UK). X-ray powder diffraction of DOX, polymers, physical mixtures and DOX-loaded NPs was carried out using an X-ray diffractometer (Rigaku Corporation, Kent, England).

### 2.4 *In vitro* antibacterial activities

#### 2.4.1 Culture of bacterial strains

The bacterial strains used were *Staphylococcus aureus* (NCTC® 10788) (SA1), *Staphylococcus aureus* (ATCC® BAA1707TM) (SA2), *Pseudomonas aeruginosa* (ATCC® 9027) (PA1) and *Pseudomonas aeruginosa* [PAO1] (ATCC®BAA-47) (PA2). All the strains were obtained from LGC Standards, Middlesex, UK, maintained at 4 °C and sub-cultured at regular intervals on fresh media. Prior to each antibacterial study, the bacterial strains were cultivated in tryptic soy broth (TSB), at 100 rpm and 37 °C overnight. The bacterial pellets were collected by centrifugation for 25 min at 3000 rpm. The formed pellet was resuspended in fresh TSB and optical density at 550 nm of the bacterial suspensions was set in order to obtain an equivalent to 1.5 × 10^8^ CFU/mL.

#### 2.4.2 Determination of minimum inhibitory concentration and minimum bactericidal concentration

The minimal inhibitory concentrations (MIC) and minimal bactericidal concentrations (MBC) of DOX, blank NPs and DOX-loaded NPs were determined by a microtiter broth dilution technique in 96-well bottom-plates as per the protocol of the Clinical and Laboratory Standards Institute (Patel J.B. et al., 2015). Briefly, 100 μL bacteria suspension (1.5 × 10^8^ CFU/mL) was cultured in a 96-well plate in their respective medium in the presence of 100 μL of different concentrations of DOX, blank NPs and DOX-loaded NPs, resulting in 2 × 10^5^ CFU/mL of bacteria. The microplates were incubated at 37 °C for 24 h. The MIC was defined as the lowest concentration of DOX, blank NPs and DOX-loaded NPs at which no visible growth of the bacteria following incubation was observed. For MBC determination, 20 μL from wells corresponding to the MIC and the above-mentioned dilutions were cultured onto TSA plates and incubated at 37 °C for 24 h. Afterwards, the bacterial colonies on the plates were counted. The lowest concentration that killed 99.9% of the bacterial growth was defined as the MBC.

#### 2.4.3 Time kill assay

Time-killing kinetics of DOX and DOX-loaded NPs against SA and PA were determined as per the method described previously (Chen et al., 2018). In brief, concentrations equal to MIC, 2 x MICs and 4 x MICs of DOX, blank NPs and DOX-loaded NPs were prepared and were added into the bacterial suspensions, resulting in 2 × 10^5^ CFU/mL of bacteria. The bacterial cultures were then incubated at 37 °C. Aliquots of 20 μL from the cultures were collected at time intervals of 0, 2, 4, 6, 8, 12, 18 and 24 h and inoculated aseptically into TSA plates. The plates were incubated at 37 °C for 24 h and the viable colony forming units (CFU) of the bacteria was determined. The procedure was carried out in triplicate and a curve of the log CFU/mL was constructed against time-kill.

### 2.5 *In-vitro* release study of DOX-loaded NPs in bacterial cultures

The release studies of free DOX and DOX-loaded NPs were carried out with or without the bacterial cultures (Xiong et al., 2012). Briefly, NPs equivalent to 5 mg of DOX were dispersed in 5 mL of the bacterial cultures (optical density at 550 nm was set to 0.1) and were incubated in an orbital shaker at 100 rpm, 37 °C. At predetermined time intervals, aliquots of 0.5 mL of sample were drawn out and were filtered using Amicon® Ultra Centrifugal Device (Millipore Inc., molecular weight cut-off (MWCO) of 12 kDa). The amount of DOX in the filtrate was quantified by HPLC.

### 2.6 *In vitro* antibiofilm activities

#### 2.6.1 96-Well Microtiter Plate (MTP) Biofilm Study

The effects of DOX, blank NPs and DOX-loaded NPs on biofilm-grown of SA and PA were carried out using the crystal violet technique (Bazargani and Rohloff, 2016). Briefly, these bacterial cells were cultivated in TSB with 3% (w/v) NaCl and 0.5% (w/v) glucose (TSB-NG) and diluted to obtain 2 × 10^5^ CFU/mL of bacteria. Aliquots of 200 μL of the bacterial suspension were then placed into a 96-well plate and were incubated for 24 h at 37 °C. Afterwards, the wells were carefully washed thrice with sterile PBS to remove any non-adherent microorganisms. The concentrations equal to MIC, 2 x MICs and 4 x MICs of DOX, blank NPs and DOX-loaded NPs (200 μL), as well as TSB-NG (200 μL) as control were then added into the preformed biofilms and were incubated for 24 h at 37 °C. Following incubation, the non-adherent bacterial cells were removed. The biofilms were rinsed thrice with 200 μl of sterile PBS. The plates were dried for 1 h at 25 °C. The biofilms in the wells were further stained with 200 μl of 1% w/v crystal violet and were incubated for 15 at room temperature. To remove any unabsorbed stains, the wells were washed three times with sterile distilled water. An aliquot of 200 μL of ethanol was added into the washed wells to dissolve the crystal violet. Finally, the destaining solutions were moved to a new plate and the absorbance, presenting the amount of biofilm, was determined at 595 nm using a FLUOstar Omega spectrophotometer (BMG LabTech, Ortenberg, Germany). The killing percentage was calculated using Eq. (3).

(3) $$\text{Killing percentage}=\frac{{\mathrm{Abs}}_{\mathrm{control}}-{\mathrm{Abs}}_{\mathrm{experimental}}}{{\mathrm{Abs}}_{\mathrm{control}}}\times 100\%$$

#### 2.6.2 Colony Biofilm Model (CBM)

The antibiofilm activities of DOX, blank NPs and DOX-loaded NPs were also evaluated in the CBM (Alhusein et al., 2016), with slight modifications. Briefly, 50 μL of the diluted bacterial 2 × 10^5^ CFU/mL were inoculated onto sterile 10 mm poly(carbonate) discs located on the surface of tryptic soy agar (TSA) plates. The plates were incubated for 72 h at 37 °C to permit the development of the biofilm. The discs were aseptically moved to new TSA plates every day for 4 days. Afterwards, 100 μL DOX, blank NPs and DOX-loaded NPs with the concentrations equal to MIC, 2 × MICs and 4 × MICs were spotted on to the discs using a micropipette. The disc without any treatments was used as a control. The plates were further incubated at 37 °C for 24 h. Following 24 h incubation, each disc was moved to a tube containing 5 mL of TSB and were vortexed for 5 min to separate the bacteria from the discs and to disrupt the biofilms. The suspended cells were then diluted appropriately and 20 μL were inoculated aseptically into TSA plates. The plates were incubated at 37 °C for 24 h and the viable CFU were calculated. The killing percentage was calculated using Eq. (4).

(4) $$\text{Killing percentage}=\frac{{\mathrm{CFU}}_{\mathrm{control}}-{\mathrm{CFU}}_{\mathrm{experimental}}}{{\mathrm{CFU}}_{\mathrm{control}}}\times 100\%$$

### 2.7 Fabrication of two-layered dissolving MNs

Two-layered dissolving MNs were prepared by a two-step casting technique (Permana et al., 2019c), with slight modifications. An aqueous blend of 25% w/w of PVP (58 kDa) and 15% of PVA (31–50 kDa) was used a MNs matrix. This aqueous blend was mixed with the same amount of washed pellets of DOX-loaded NPs. Following this, 50 mg of the formulation was cast into the silicone MN moulds (needle density of 16 × 16, pyramidal needles; 850 μm height [600 μm pyramidal tip, 250 μm base column] and 300 μm width at base and 300 μm interspacing). The moulds were further placed in a positive pressure chamber and a pressure of 4 bar was applied for 2 min. The excess formulations on the mould were removed using a spatula and the formulations were dried for 4 h at room temperature. Afterwards, an aqueous gels of 15% w/w PVP (360 kDa) and 1.5% w/w glycerol was then poured on the top of dry formulation, forming the two-layered MNs. The moulds were placed again in a positive pressure chamber and a pressure of 4 bar was applied for 15 min. The MNs were finally dried at room temperature for 48 h. In addition to MNs containing DOX-loaded NPs, MNs containing free DOX were also prepared for further studies using the same method and the same DOX concentration. The morphology of MNs were visually examined using a Leica EZ4D light microscope (Leica Microscope, Milton Keynes, UK) and scanning electron microscope (SEM) TM3030 (Hitachi, Krefeld, Germany).

### 2.8 Evaluation of mechanical and insertion properties of dissolving MNs

Mechanical strength of MNs was evaluated using a TA-TX2 Texture Analyzer (Stable Microsystem, Haslmere, UK), as described previously (González-Vázquez et al., 2017). The insertion capability of MNs was investigated using Parafilm®M as a validated skin-simulant artificial membrane, as described in a previous study (Larrañeta et al., 2014). Additionally, the insertion property of MNs was also investigated using an optical coherence tomography (OCT) microscope (Michelson Diagnostics Ltd., Kent, UK), as outlined in previous works (González-Vázquez et al., 2017; McCrudden et al., 2018; Permana et al., 2019a), in Parafilm® M and full-thickness neonatal porcine skin. ImageJ® (National Institute of Health, Bethesda, MD, USA) software was employed to measure the length of needles inserted.

### 2.9 Calculation of drug content localized to the needles

To quantify the amount of DOX in MN needles, the needles were detached cautiously from the baseplate using a scalpel and were dissolved in 5 mL distilled water. The drug amount was calculated using the same protocol as described in drug loading calculation in NPs formulation.

### 2.10 Investigation of the effect of MN formulation on the size and PDI of SLNs

The two-layered dissolving MNs were dissolved in distilled water. Afterwards, the size and PDI of the NPs were measured using DLS and were compared to the size and PDI of NPs before being formulated into MNs arrays.

### 2.11 Dissolution study, *ex vivo* dermatokinetic studies and antibiofilm activity in *ex vivo* model of biofilm on porcine skin

#### 2.11.1 Preparation of *ex vivo* model of biofilm on porcine skin

Excised full-thickness skin from stillborn piglets was obtained as described previously (Permana et al., 2019a). The skins were sterilised by immersing in 70% ethanol for 1 h and were dried for 20 min in a biosafety cabinet. *Ex vivo* models of biofilm in the excised full-thickness neonatal porcine skin were created by following the methods described previously (Alhusein et al., 2016), with minor modifications. Briefly, two types of wound were created, namely using a biopsy punch (Stiefel, Middlesex, UK) and red-hot brass knob, forming cut wounds (wound 1) and burn wounds (wound 2) of diameter 5 mm, respectively. The skin pieces were aseptically placed on TSA plates in Class II Microbiological safety cabinet using a metal tweezer. Afterwards, 50 μL of the diluted bacterial suspensions 2 × 10^5^ CFU/mL were inoculated to the wound of the skin and spread homogeneously. The plates were incubated at 37 °C with the skin were aseptically transferred to new TSA plates every day for 5 days to allow the formation of the biofilm. The biofilm formation was observed using a a Leica EZ4D light microscope and OCT.

#### 2.11.2 Dissolution study

The MNs dissolution was considered *in situ* in *ex vivo model of biofilm on porcine skin*. Briefly, the MN arrays were then inserted into the skin section using manual pressure and a cylindrical stainless-steel weight of 5.0 g put on top to assure the array remained in the same place. At different interval time points, MN arrays were removed and viewed under a Leica EZ4 D stereo microscope.

#### 2.11.3 *Ex vivo* dermatokinetic studies

*Ex vivo* dermatokientic studies of dissolving MNs containing free-DOX and DOX-loaded NPs were conducted in uninfected and biofilm model on excised full-thickness porcine skin. This study was carried out as per a method described previously (Permana et al., 2019a, Permana et al., 2019c), Initially, the skin was attached to the donor compartment of the Franz cell diffusion cells using a cyanoacrylate glue. The MNs were inserted into the skin for 30 s using manual force and the donor compartment was connected to the receiver compartment containing PBS (pH 7.4). To ensure the MNs stayed in place during the experiment, a cylindrical stainless-steel mass of 5 g was placed on top of the MNs. Parafilm®M was used to seal the donor compartment and sampling arm and the temperature of the receiver compartment was maintained at 37 ± 1 °C. The compartment was stirred at 600 rpm. At predetermined time points (0.5 h, 1 h, 2 h, 3 h, 4 h, 5 h, 6 h, 8 h, 12 h, 24 h and 48 h), the MNs were removed and the skin was rinsed thrice with sterile water. To extract the DOX released in the skin, 1.5 mL water and two stainless steel beads (5 mm diameter) were added along with the skin to Eppendorf tubes and the mixture was homogenized for 15 min at 50 Hz, using a Tissue Lyser LT (Qiagen, Ltd., Manchester, UK). The samples were then centrifuged at 14,000 rpm for 30 min and the amount of DOX in supernatant was quantified by HPLC. PKSolver (China Pharmaceutical University, Nanjing, China) (Zhang et al., 2010) was applied in a one-compartment open model to evaluate the dermatokinetic profiles. The maximum drug concentration in skin (Cmax), the time of maximum concentration (t_max_), the drug concentration time curve from time zero (*t* = 0) to the last experimental time point (*t* = 72 h) (AUC), the mean half-life (t_1/2_) and the mean residence time (MRT) were all determined. In order to ensure that the extraction method was only able to extract the DOX released from NPs without disrupting the NPs, the two stainless steel beads were also separated added into DOX-loaded NPs dispersion and processed as the same procedure as the skin extraction. As a comparison, needle-free patches containing free-DOX and selected DOX-loaded NPs were prepared and the same studies were carried out.

#### 2.11.4 Antibiofilm activity in *ex vivo* model of biofilm on porcine skin

Antibiofilm activity was sudied using the method described previously (Alhusein et al., 2016; Roche et al., 2019), with slight modifications. To evaluate the antibiofilm activity of dissolving MNs and needle-free patches containing free-DOX and DOX-loaded NPs in *ex vivo* model, 20 μL of the supernatant collected from dermatokinetic studies following 24 h and 48 h application time were inoculated into TSA plates and were incubated for 24 h at 37 °C. Additionally, blank dissolving MNs were also applied to the infected skin and the same procedure was performed. The numbers of viable CFU were finally counted. Infected skin without MNs application were used as a positive control and normal skin were used as a negative control.

### 2.12 Instrumentation and chromatographic condition for analytical method

The concentrations of DOX in all studies were quantified by HPLC (Agilent Technologies 1220 Infinity UK Ltd., Stockport, UK) as described earlier (Permana et al., 2019b), with slight modifications. The quantifications were performed using a Xselect CSH™ C18 column (Waters, 3.0 × 150 mm with particle sizes of 3.5 μm) with the flow rate of 0.4 mL/min. A mixture of 0.1% v/v of trifluoroacetic acid in water and acetonitrile (65:35 v/v) was used as the mobile phase and UV detector was set at 270 nm. The injection volume was 25 μL and the quantification were conducted at room temperature. This analytical method was validated according to the International Committee on Harmonisation (ICH) guidelines 2005.

### 2.13 Statistical analysis

The experimental results were reported as means ± standard deviation (SD) of the mean. The results were statistically analyzed using GraphPad Prism® version 6 (GraphPad Software, San Diego, California, USA). An unpaired *t*-test was used to compare two groups. The Kruskal-Wallis test with post-hoc Dunn's test was used to compare multiple groups. A value *p* < .05 denoted statistical significance.

## 3 Results and discussion

### 3.1 Formulation of DOX-loaded NPs

DOX-loaded PLGA and PCL were prepared a using double emulsion method because this method is an appropriate method to formulate NPs containing hydrophilic molecules (Cohen-Sela et al., 2009), like DOX. For chitosan NPs, the formulations were prepared using the ionotropic gelation between positively charged chitosan with negatively charged TPP (de Carvalho et al., 2019). The characteristics of NPs are shown in Table 2. The results showed that the particle sizes of NPs were found to be 247.5 ± 12.5 nm, 208.3 ± 9.8 nm, 262.6 ± 21.4 nm, 235.1 ± 11.2 nm and 217.4 ± 10.3 nm for NP-1, NP-2, NP-3, NP-4 and NP-5, respectively. It was observed that NPs obtained from PCL (NP-2) were significantly smaller (*p* < .05) compared to PLGA NPs (NP-1). It has been postulated that PCL chains possess higher flexibility than PLGA chains and, therefore, the formulation of drugs into this polymer could potentially result in smaller particle sizes (Mundargi et al., 2007). When compared to chitosan NPs (NP-5), the particle sizes of NP-1 were found to significantly greater (*p* < .05). This may be due to the hydrophobicity of PLGA in comparison to chitosan, which could potentially affect the particle size in NP formulations. For NP-3 and NP-4, the particle sizes were greater compared to NP-1 and NP-2 most likely because of the coating process of chitosan onto the surface of PLGA and PCL NPs. Specifically, the incorporation chitosan into PCL NPs did not increase the particle size significantly (*p* > .05). With respect to PDI values, it was found that the values were in the range of 0.198 ± 0.004 and 0.232 ± 0.003, demonstrating the generally homogeneous and monodispersed profile of these NPs.

**Table 2:** Particle size, PDI, zeta potential, encapsulation efficiency and drug loading capacity of different formulations of DOX-loaded NPs (means ± SD, *n* = 3).

Formulations	Particle size (nm)	PDI	Zeta potential (mV)	EE (%)	DL (%)
NP-1	247.5 ± 12.5	0.201 ± 0.011	−6.3 ± 0.58	50.5 ± 3.4	11.9 ± 1.2
NP-2	208.3 ± 9.8	0.198 ± 0.004	−7.2 ± 0.45	43.4 ± 4.9	15.7 ± 1.3
NP-3	262.6 ± 21.4	0.213 ± 0.013	20.4 ± 1.23	51.6 ± 5.2	9.5 ± 0.8
NP-4	235.1 ± 11.2	0.232 ± 0.003	19.2 ± 0.87	42.9 ± 4.8	12.4 ± 1.1
NP-5	207.4 ± 10.3	0.215 ± 0.009	29.5 ± 1.34	53.2 ± 4.3	11.4 ± 0.9

For zeta potential, the values were −6.3 ± 0.58 mV for NP-1, −7.2 ± 0.45 mV for NP-2, 20.4 ± 1.23 mV for NP-3, 19.2 ± 0.87 mV for NP-4 and 29.5 ± 1.34 mV for NP-5. The negative zeta potentials obtained from NP-1 and NP-2 were as a result of the presence of ionized of carboxylic group of PLGA and PCL (Öcal et al., 2014). In contrast, a positive zeta potential was achieved in the case of NPs with chitosan. This is most likely due to the presence of positive amine groups in the chitosan (Rabea et al., 2003), leading to the positively charged NPs.

Regarding the EE, it was found that the EEs of NPs prepared from PCL (NP-2 and NP-4) were significantly smaller (*p* < .05) compared to PLGA NPs (NP-1 and NP-3) and chitosan NPs (NP-5). The lower EE may be due to the higher hydrophobicity nature of PCL (Badran et al., 2018) in comparison with PLGA and chitosan. Therefore, the encapsulation of DOX as a hydrophilic compound were low in this polymer. The EE values were 50.5 ± 3.4%, 43.4 ± 4.9%, 51.6 ± 5.2%, 42.9 ± 4.8% and 53.2 ± 4.3% for NP-1, NP-2, NP-3, NP-4 and NP-5, respectively. In terms of DL, the values were between 9.5 ± 0.8% and 15.7 ± 1.3%. After the attachment of chitosan to the surface of PLGA and PLGA NPs, the DLs of these NPs (NP-3 and NP-4) decreased from 11.9 ± 1.2% to 9.5 ± 0.8 and from 15.7 ± 1.3% to 12.4 ± 1.1%. This was caused by the additional amount of NPs composition, while maintaining the amount of DOX in the NPs, leading to the decreasing DL value.

**Fig. 1:**
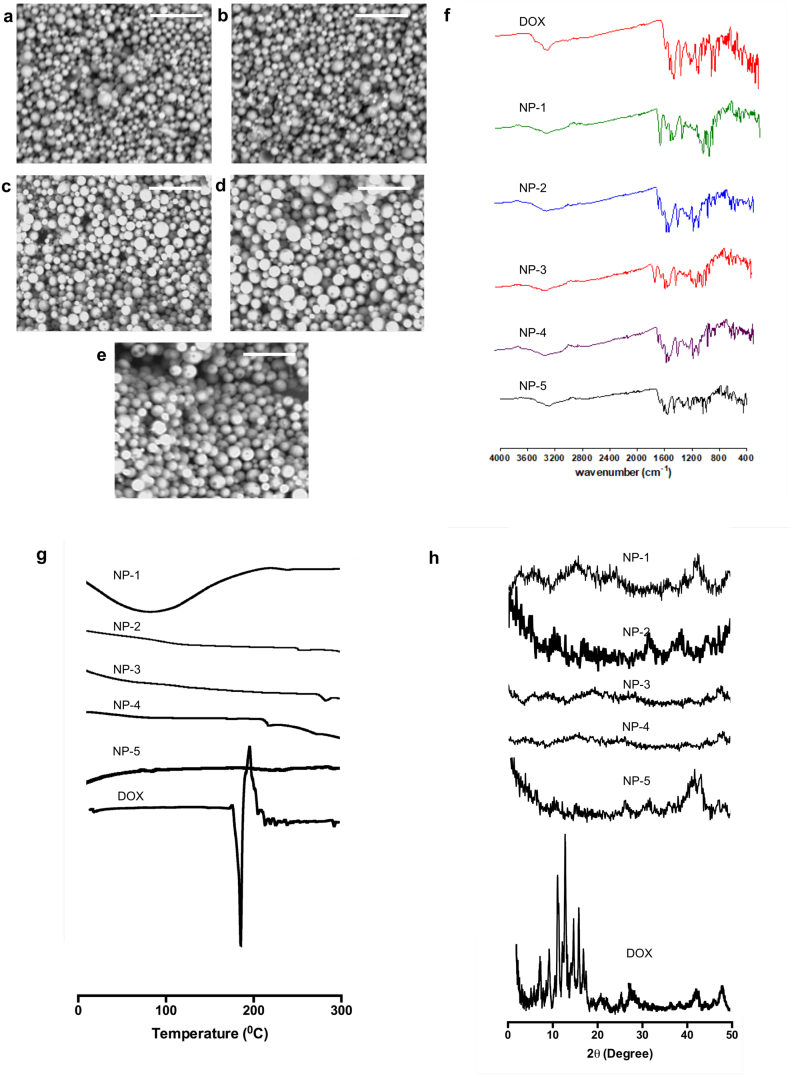
SEM images of NP-1 (a), NP-2 (b), NP-3 (c), NP-4 (d) and NP-5 (e) at a magnification power of 30,000× (The white scale bar represents a length of 1 μm in each case). FTIR spectra of DOX, NP-1, NP-2, NP-3, NP-4 and NP-5 (f). DSC thermogram of DOX, NP-1, NP-2, NP-3, NP-4 and NP-5 (g). X-ray diffractogram of DOX, NP-1, NP-2, NP-3, NP-4 and NP-5 (h).

The morphologies of DOX-loaded NPs observed by SEM are depicted in Fig. 1a–e. As shown in SEM images, all formulations were approximately spherical in shape. Importantly, the sizes obtained in this observation were in close agreement with the results obtained from DLS analysis (˜200 nm).

FTIR spectra of DOX and their respective NPs formulations are depicted in Fig. 1f. The spectra indicate that DOX had a major peak at 1518 cm^−1^ due to aromatic N—H bending. Specific peaks were also observed between 1341 cm^−1^ and 1679 cm^−1^ due to aromatic C=O and C=C stretching. Peaks were found at 2921 cm^−1^, 3311 cm^−1^ and 3432 cm^−1^ relating to C—H stretching, primary N—H group and primary O—H group, respectively. Importantly, specific groups were also observed in FTIR spectra of all NPs formulations. Accordingly, chemical interactions between excipients used and DOX were not shown to occur.

The DSC analysis results of DOX and DOX-loaded NPs are shown in Fig. 1g. The results showed sharp endothermic peaks at 178 °C in DOX, owing to the melting points of the DOX crystals. Nevertheless, this peak was not observed in DOX-loaded NPs. The XRD diffractograms of the pure DOX and its NP formulations are depicted in Fig. 1h. Sharp characteristics peaks of DOX were observed at 2θ values of 10.42, 15.21, 17.71 and 19.98, because of high crystalline characteristics of DOX, as reported previously (Eskitoros-Togay et al., 2019). However, similar to DSC analysis results, these peaks were not observed in NP formulations. The absence DOX peak in DSC and XRD analysis may be caused by the complete encapsulation of DOX in amorphous or solution form in the NP polymers used in this study.

### 3.2 *In vitro* antibacterial activities

#### 3.2.1 Determination of minimum inhibitory concentration and minimum bactericidal concentration

The antibacterial activity of free DOX and DOX-loaded NP formulations were further investigated. The comparison of MIC and MBC values between DOX solution and their NP formulations are presented in Table 3. The results implied that the formulation of DOX into NPs enhanced the antibacterial activity of DOX against SA and PA strains. Specifically, the NPs prepared from chitosan (NP-3, NP-4 and NP-5) exhibited lower MIC and MBC, compared to the NPs without chitosan. This was a result of the utilization of chitosan in our formulation. In the blank NPs, despite higher MIC and MBC obtained in our study, it was found that the blank NPs containing chitosan showed antimicrobial activities against all bacterial strains. Similarly, it has been well reported that chitosan has an antibacterial activity (Choi et al., 2019; Noel et al., 2010; Rabea et al., 2003). In this study, all MBC values were higher than MIC, indicating that higher DOX concentration was required to kill the bacterial cultures. Comparing the ratio of MBC to MIC, it was found that the ratio of these values in all case was <4. The ratio of ≤4 is denoted as bactericidal and the ratio of >4 is denoted as bacteriostatic (Tato et al., 2014). Accordingly, our study suggested that both DOX and DOX-loaded NPs possessed bactericidal activities.

**Table 3:** MIC and MBC values of free DOX, blank NPs and DOX-loaded NPs (*n* = 3).

		Bacterial strains							
		SA1		SA2		PA1		PA2	
		DOX-loaded NPs	Blank NPs	DOX-loaded NPs	Blank NPs	DOX-loaded NPs	Blank NPs	DOX-loaded NPs	Blank NPs
MIC (μg/mL)	Free DOX	12.5	>12,800	25	>12,800	25	>12,800	50	>12,800
	NP1	6.25	>12,800	12.5	>12,800	12.5	>12,800	25	>12,800
	NP2	6.25	>12,800	12.5	>12,800	12.5	>12,800	25	>12,800
	NP3	3.125	400	6.25	800	6.25	400	12.5	3200
	NP4	3.125	400	6.25	800	6.25	400	12.5	3200
	NP5	3.125	200	3.125	400	3.125	200	6.25	1600
MBC (μg/mL)	Free DOX	25	>12,800	50	>12,800	25	>12,800	100	>12,800
	NP1	12.5	>12,800	25	>12,800	25	>12,800	50	>12,800
	NP2	12.5	>12,800	25	>12,800	25	>12,800	50	>12,800
	NP3	6.25	800	12.5	1600	12.5	1600	25	6400
	NP4	6.25	800	12.5	1600	12.5	1600	25	6400
	NP5	6.25	400	12.5	800	6.25	800	25	3200

#### 3.2.2 Time kill assay

**Fig. 2:**
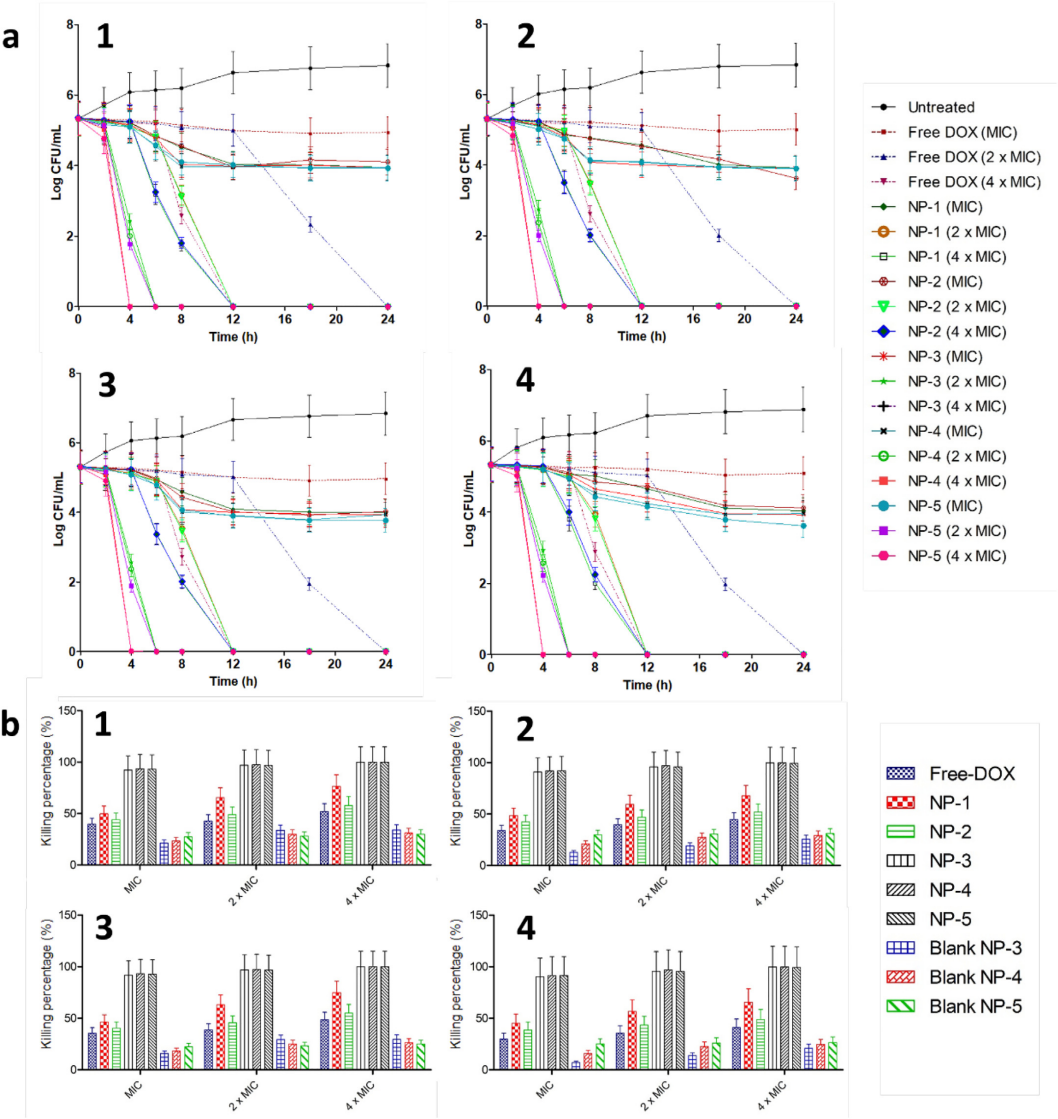
Time kill assay of DOX and DOX-loaded NPs (a) against *Staphylococcus aureus* (NCTC® 10,788) (SA1) (1), *Staphylococcus aureus* (ATCC® BAA1707TM) (SA2) (2), *Pseudomonas aeruginosa* (ATCC® 9027) (PA1) (3) and *Pseudomonas aeruginosa* [PAO1] (ATCC®BAA- 47) (PA2) (4) (means ± SD, *n* = 3). Antibiofilm activity in 96-well microtiter plate of DOX, DOX-loaded NPs and blank NPs (b) against *Staphylococcus aureus* (NCTC® 10,788) (SA1) (1), *Staphylococcus aureus* (ATCC® BAA1707TM) (SA2) (2), *Pseudomonas aeruginosa* (ATCC® 9027) (PA1) (3) and *Pseudomonas aeruginosa* [PAO1] (ATCC®BAA- 47) (PA2) (4) (means ± SD, *n* = 3).

In an attempt to explain the time required by DOX and DOX-loaded NPs to kill the bacterial strains tested, time kill assays were carried out. Fig. 2a displays the time kill curve of free DOX and DOX-loaded NPs against four bacterial strains. In the untreated cohort, the viable bacterial counts increased by around 6.8 log CFU following 24 h incubation time in all bacterial strains. With respect to the concentration tested, DOX and DOX-loaded NPs with MIC values were not able to kill 99.99% of bacteria after 24 h. With 2 times the MIC values, no bacteria were counted in the case of free DOX after 24 h. Interestingly, at this concentration, 100% of bacteria were killed after 12 h for NP-1 and NP-2, after 12 h for NP-3 and NP-4 and after 6 h for NP-5. Similar results were also found in the concentration of 4 times of the MIC of NP-1 and NP-2. On the other hand, the killing time decreased to 4 h for NP-3, NP-4 and NP-5 after incubation with 4 times the MIC values. The results obtained here imply that the killing rates of DOX and DOX-loaded NPs were concentration dependently. The formulation of DOX into NPs was not only able to increase antibacterial activity, indicated by the lower MIC presented previously, but also decrease the time required to kill the bacteria.

### 3.3 *In vitro* release study of DOX-loaded NPs in bacterial cultures

**Fig. 3:**
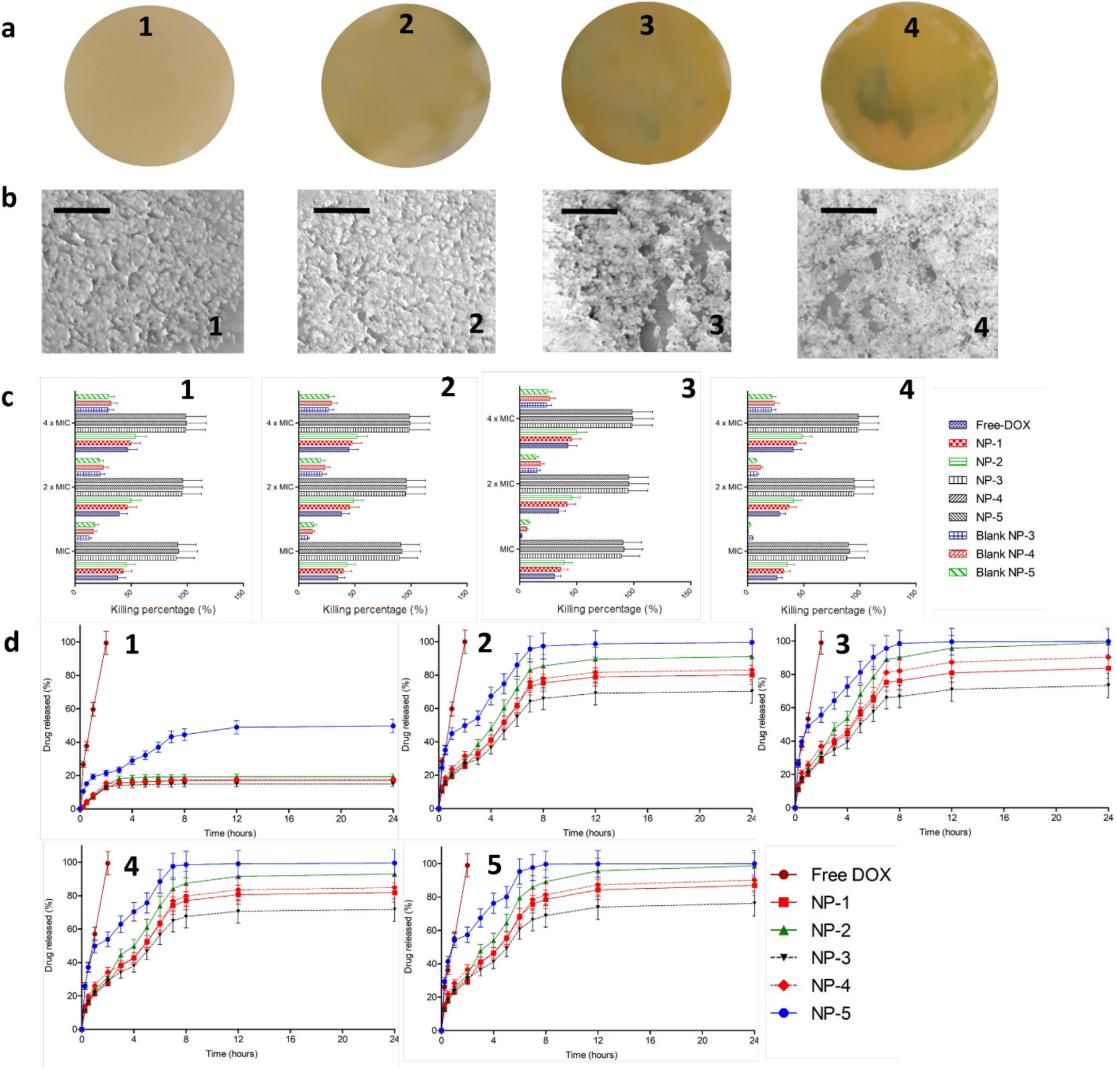
Representative images of colony biofilm models (CBM) (a) of SA1 (1), (SA2) (2), (PA1) (3) and (PA2) (4). Representative SEM images of bacterial biofilms models (b) of SA1 (1), (SA2) (2), (PA1) (3) and (PA2) (4) (The black scale bar represents a length of 10 μm in each case). Antibiofilm activity of DOX, DOX-loaded NPs and blank NPs in CBM (c) against SA1 (1), (SA2) (2), (PA1) (3) and (PA2) (4) (means ± SD, *n* = 3). *In-vitro* drug release from NPs in the absence of bacterial culture (1) and presence of SA1 (2), (SA2) (3), (PA1) (4) and (PA2) (5) (mean ± SD, n = 3).

This study was designed to develop NP formulations containing DOX for specific delivery to infected site. Thus, the release behaviors of DOX from its NPs formulations with and without the bacterial cultures were then investigated. Two different strains of SA and PA as above-mentioned were selected. Fig. 3d depicts the cumulative percentage release of DOX from NP-1, NP-2, NP-3, NP-4 and NP5 in sterile media and media containing SA1, SA2, PA1 and P4, in comparison with DOX solution. As shown in Fig. 3d, the presence of bacterial cultures significantly improved (*p* < .05) the release of DOX from NPs, compared to the release profiles in the absence of bacterial cultures, indicating the successful on demand delivery of these formulations. The presence of bacterial cultures did not affect (*p* > .05) the release behavior of DOX solutions (approximately 100% of drug released after 2 h). Specifically, after 24 h, in the absence of bacteria cultures, the release percentage of DOX were found to be 19.33 ± 1.54%, 17.03 ± 1.19%, 17.63 ± 1.41%, 14.91 ± 1.32% and 49.74 ± 3.97% from NP-1, NP-2, NP-3, NP-4 and NP-5, respectively. In the presence of SA1, 80.22 ± 5.61%, 91.06 ± 7.29%, 70.73 ± 6.98%, 83.05 ± 6.64% and 97.56 ± 8.76% of DOX were released from NP-1, NP-2, NP-3, NP-4 and NP-5, respectively. With respect to the release profile of DOX in SA2 cultures, the total release percentage of DOX were 81.96 ± 7.55% from NP-1, 93.03 ± 8.98% from NP-2, 71.79 ± 7.09% from NP-3, 84.58 ± 6.98% from NP-4 and 98.67 ± 7.98% from NP-5. In PA1 cultures, the percentages of 86.99 ± 7.93%, 98.76 ± 8.03%, 76.21 ± 7.12%, 90.06 ± 8.98% and 99.01 ± 9.82 of DOX were found to be released from NP-1, NP-2, NP-3, NP-4 and NP-5, respectively. Following 24 h incubation period with PA2, the highest percentage of DOX released from all NPs were achieved, which were 83.77 ± 5.68%, 99.43 ± 7.43%, 73.5 ± 6.98%, 94.54 ± 10.42% and 99.43 ± 7,43% from NP-1, NP-2, NP-3, NP-4 and NP-5, respectively.

The results obtained here showed the selective release profile of DOX from DOX-loaded NPs with the presence of bacterial cultures. Therefore, this approach could be potentially beneficial for permitting delivery only at the infection site, avoiding the non-specific release to uninfected areas. The difference of release behavior might be caused by the selection of polymers in the NPs formulations. It has been previously reported that, due to the rapid degradation of PCL by lipase enzymes secreted by SA, the release of vancomycin from PCL nanogels in the presence of this bacterial was significantly improved compared to the release profile without the bacteria (Xiong et al., 2012). Additionally, the release of fusidic acid from PLGA ultrafine fibers was also significantly enhanced in the presence of SA and PA (Said et al., 2011). Chitosan has been also reported to be hydrolyzed by the lipase enzyme (Chiou and Wu, 2004). Comparing the release profile of all NPs, it was shown that, despite non-significant differences (*p* > .05), the amount of DOX released from NP-2 was lower than NP-1 in all bacterial cultures. The higher release from NP-1 might be caused by the greater flexibility of the PCL chains compared to the PLGA chains (Badran et al., 2018), leading to the rapid hydrolysis by lipase esterase secreted by the bacterial cultures in the media. The presence of chitosan on the surface of these NPs insignificantly decreased (*p* > .05) the release of DOX. Hence, we successfully developed NPs laden with DOX for bacteria sensitive release.

### 3.4 *In vitro* antibiofilm activities

#### 3.4.1 96-Well microtiter plate (MTP) biofilm study

In burn and chronic wounds, the development of biofilm results in significant challenge in the effectiveness of antimicrobial therapy. In this study, we evaluated the ability of DOX-loaded NPs to kill the bacterial biofilms, in comparison with free DOX and blank NPs. Fig. 2b exhibits the percentage of reduction of bacterial biofilm after the application of DOX, DOX-loaded NPs and blank NPs. For the blank NPs, as blank NP-1 and blank NP-2 did not show antimicrobial activities, the antibiofilm activities of these blank NPs were not carried out. Free DOX was able to kill only between 29.68 ± 3.12% and 51.75% of biofilm cells in all bacterial strains in all DOX concentrations. In the concentration of four-fold of MIC, NP-1 was able to kill 76.39 ± 12.32% of SA1, 67.73 ± 10.23% of SA2, 74.81 ± 11.98% of PA1 and 65.18 ± 9.34% of PA1. With respect to NP-2, 57.43 ± 10.02% of SA1, 51.88 ± 8.43% of SA2, 55.02 ± 6.54% of PA1 and 48.76 ± 8.21% of PA2 were killed following incubation in the concentration of four times of MIC. Interestingly, in the case of NP-3, NP-4 and NP-5, more than 90% of bacterial biofilms were killed after incubation in these NPs with the MIC values. More than 99% of bacterial biofilms were killed when four-fold of MIC were incubated with the bacterial biofilms. In terms of the blank NPs, due to the presence of chitosan in the formulations, blank NP-3, blank NP-4 and blank NP-5 exhibited antibiofilm activities with around 20.75 ± 3.13%–34.03 ± 5.43% of biofilms were killed after incubation in the concentration of four-fold of MIC. Analyzed statistically, NP-3, NP-4 and NP-5 were able to decrease the viability of bacterial biofilms significantly (*p* < .05) in comparison with other formulations. With these results, it was observed that the presence of chitosan in the NPs surface influenced the ability of NPs to kill bacterial biofilms. The presence of bacterial biofilms has been reported to increase resistance of bacterial to antibacterial agents (Høiby et al., 2010). In addition, conventional antibiotics have shown poor ability to penetrate bacterial biofilms. This may be related to the presence of a dense barrier formed by the biofilm matrix components limiting the penetration of antibiotic agents (Smith, 2005; Szomolay et al., 2005). Bacterial biofilms have the net negative charge due to the presence of EPS. Therefore, the positive charge NPs might be easily attached to negative charge of bacterial biofilm, enabling the penetration of antibacterial agents into the bacterial cells (Han et al., 2017). Despite the fact that all DOX-loaded NPs were able to kill the planktonic bacteria, not all formulations showed efficacy against bacterial biofilms. We showed that only positively charged DOX-loaded NPs (NP-3, NP-4 and NP-5) exhibited effective killing percentage to the bacterial biofilms. Our results are supported by a previous study carried out by Hasan et al., reporting that positively charged clindamycin-loaded PLGA-polyethylenimine (PLGA-PEI) NPs increased the adhesion of NPs to bacteria compared to negatively charged NPs (Hasan et al., 2019). Therefore, the decoration of NPs with chitosan provides two main advantages. Firstly, the utilization of chitosan enhanced the antibiofilm activity of DOX. Secondly, the positive charge of chitosan enabled the adhesion of NPs to the bacterial biofilms.

#### 3.4.2 Colony biofilm model (CBM)

To further exploit the ability of DOX-loaded NPs to eliminate bacterial biofilms, a colony biofilm model (CBM) was developed. In this model, a poly(carbonate) membrane was utilized as a substrate for the growth of biofilms. The membrane was placed on top of an agar plate and the mature biofilm growing in this membrane stimulates biofilm development in a wound (Alhusein et al., 2016; Anderl et al., 2000). Additionally, the small fluid shear and the closeness to an air boundary made by this system closely approximates the environment of wounds (Folsom et al., 2011). The carbon and nitrogen source of the agar are similar to the nutrient flow in biofilms of a wound (Alhusein et al., 2016). Importantly, as the membrane can be easily removed from the agar, this model allows us to count the viable bacteria by resuspending the membrane in broth medium. Representative images of this model are shown in Fig. 3a and b. In this model, similar trends to the MTP study were found. More 90% of bacterial biofilms were killed after 24 h incubation in NP-3, NP-4 and NP-5 with MIC values (Fig. 3c). More than 95% and more than 99% of bacterial biofilms were killed following 24 incubation time in the concentration of two-fold and four-fold of MIC, respectively. These values were significantly higher (*p* < .05) than the killing percentage of free DOX, NP-1, NP-2 and blank NPs. Accordingly, these results showed the effectiveness of PLGA and PCL NPs coated with chitosan in killing the mature bacterial biofilms.

### 3.5 Fabrication of two-layered dissolving MNs

**Fig. 4:**
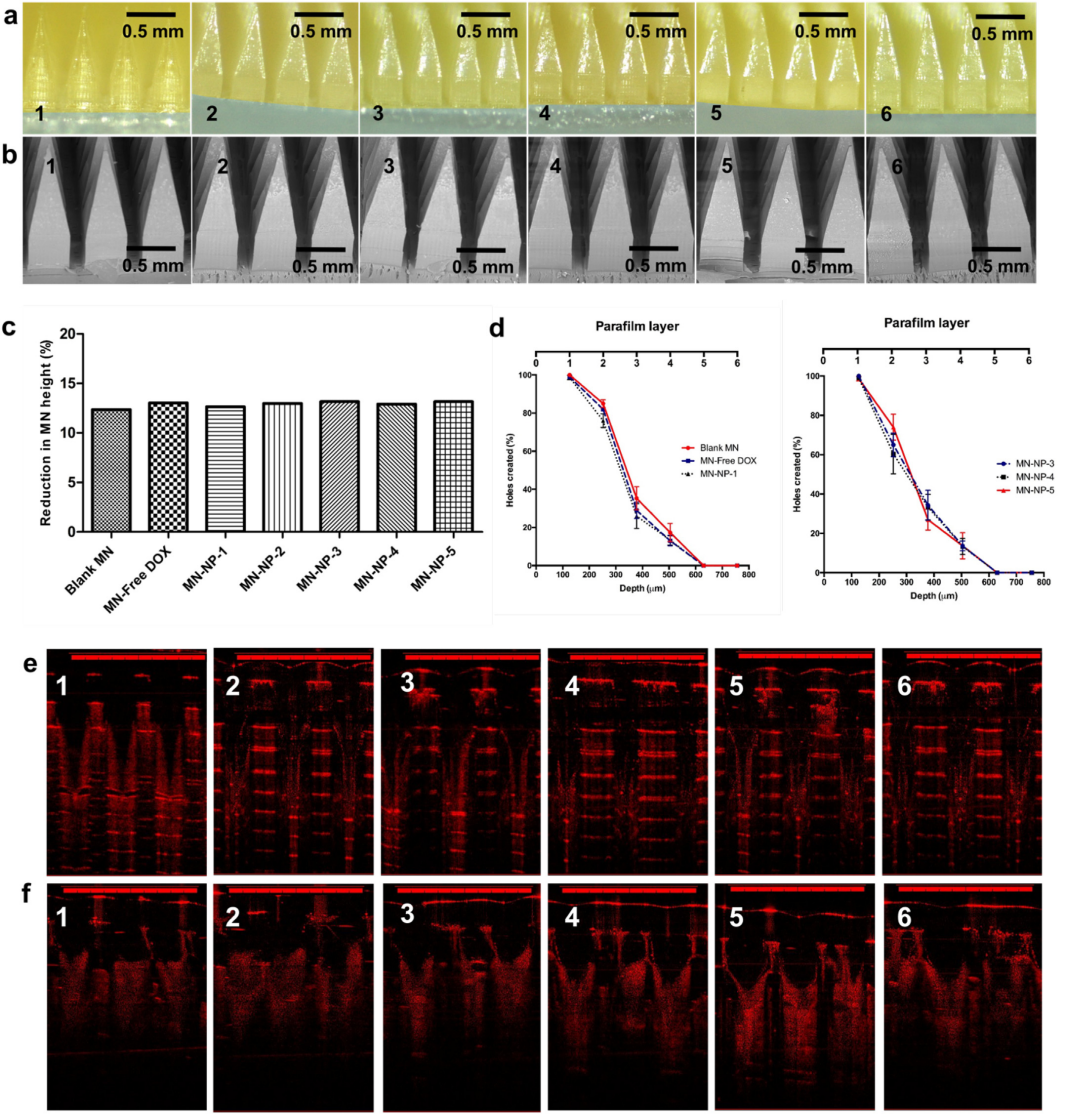
Light microscope images (a) of the MN formulations containing free DOX (1), NP-1 (2), NP-2 (3), NP-3 (4) and NP-4 (5) and NP-5 (6). SEM images (b) of the MNs containing free DOX (1), NP-1 (2), NP-2 (3), NP-3 (4) and NP-4 (5) and NP-5 (6). The percentage height reduction of needles on the arrays formulated containing free DOX and DOX-loaded NPs compared to blank MN arrays (means ± SD, *n* = 3) (c). Percentage of holes created in Parafilm®M layers, using an insertion force of 32 N/array for MN formulations containing free DOX and DOX-loaded NPs compared to blank MN arrays (means ± SD, *n* = 3) (d). Representative OCT images of MNs containing free DOX (1), NP-1 (2), NP-2 (3), NP-3 (4) and NP-4 (5) and NP-5 (6) following insertion into Parafilm®M film (e). Representative OCT images of MNs containing free DOX (1), NP-1 (2), NP-2 (3), NP-3 (4) and NP-4 (5) and NP-5 (6) following insertion into full-thickness porcine skin (f). The scale bar represents a length of 1 mm in each case.

After biofilm formation, an extracellular polymeric substance is produced, hampering the efficient delivery of antibacterial agents. In an attempt to attain biofilm disturbance and enhance the penetration of antibiotics into the biofilm, the DOX-loaded NPs were further incorporated into MN arrays. The MNs were prepared using the combination of two water soluble polymers, namely PVP and PVA. The combination of these polymers could potentially result in MNs with excellent mechanical properties, because of the hydrogen bond interaction between C=O groups of PVP and —OH groups of PVA (Permana et al., 2019c). Fig. 4a and b show the morphology of MNs of free DOX and DOX-loaded NPs observed by light microscope and SEM. The results exhibited that all MNs produced had sharp needles. Accordingly, these formulations were further characterized for their mechanical and insertion properties.

### 3.6 Evaluation of mechanical and insertion properties of dissolving MNs

The MN arrays were evaluated for their mechanical strength. This evaluation was carried out to ensure the strengthen of MN arrays in resisting compression. Importantly, the capability of MNs to be inserted in the skin is a critical parameter in MNs administration, since the needle should be able to penetrate the *stratum corneum*, to release the payload in the viable skin layers. In this study, the percentage of height reduction of MN needles after the application of 32 N/MN array, equivalent to human manual compression pressure (Larrañeta et al., 2014), was calculated to determine the mechanical strength. Fig. 4c depicts the mechanical properties of all formulations, represented by the percentage of reduction of MNs height. In this study, we compared these formulations with the blank MNs. The reductions in MN height were found to be 12.36 ± 3.12%, 13.03 ± 2.71%, 12.65 ± 3.22%, 12.98 ± 2.09%, 13.16 ± 3.10%, 12.91 ± 2.98% and 13.21 ± 2.11% for blank MNs, MN-free DOX, MN-NP-1, MN-NP-2, MN-NP-3, MN-NP-4 and MN-NP-5, respectively. Analyzed statistically, there were no significant differences in between the percentage of reduction of the MNs height of DOX and DOX-loaded NPs, compared to the blank MNs. As such, the formulation of DOX and DOX-loaded NPs into the dissolving MNs did not affect the mechanical properties of the dissolving MNs arrays.

For the insertion properties, eight-layers of Parafilm®M was utilized as a skin stimulant. This model has been previously validated to mimic human skin for MN insertion studies (Larrañeta et al., 2014). The result of this study is presented in Fig. 4d. Similar to the mechanical properties, the formulation of DOX and DOX-loaded NPs did not affect (*p* > .05) the insertion properties of any of the MNs, when compared to blank MNs. The MN arrays were able to penetrate four layers of Parafilm®M. As the mean thickness of each layer of Parafilm®M is 126 μm, then the MNs were inserted up to 504 μm, indicating that around 53.9% of the needle lengths were successfully inserted. Additionally, in an attempt to envisage the profile of insertion of the MNs, optical coherence tomography (OCT) was then utilized using the Parafilm®M and the full-thickness neonatal porcine skin models. This technique has been successfully used to visualize the insertion of ability of numerous MN formulations (Donnelly et al., 2014, Donnelly et al., 2010; González-Vázquez et al., 2017; Lutton et al., 2015; McCrudden et al., 2014; Vora et al., 2018). Fig. 4d and f depict the OCT visualization of the MNs insertion into the Parafilm®M and the full-thickness neonatal porcine skin, respectively. Analyzed using ImageJ®, the penetration depth of MNs into Parafilm®M were recorded to be 502.12 ± 21.32 μm for blank MN, 498.98 ± 19.32 μm for MN-free DOX, 505.87 ± 33.09 μm for MN-NP-1, 504.02 ± 14.54 μm for MN-NP-2, 501.16 ± 22.18 μm for MN-NP-3, 506.98 ± 29 μm for MN-NP-4 and 500.65 ± 20.32 μm for MN-NP-5. In the full-thickness porcine skin, the penetration depth of 503.65 ± 12.43 μm, 500.43 ± 10.12 μm, 506.43 ± 21.11 μm, 498.43 ± 10.41 μm, 502.11 ± 10.03 μm and 499.87 ± 10.03 μm for blank MN, MN-free DOX, MN-NP-1, MN-NP-2, MN-NP-3, MN-NP-4 and MN-NP-5, respectively. These values were in close agreement with those obtained in the insertion study according to percentages of holes created. Analyzed statistically, there were no significant differences (*p* > .05) in the penetration depth of all MNs. Hence, the incorporation of DOX and DOX-loaded NPs into dissolving MNs did not alter the penetration ability of MN arrays.

### 3.7 Calculation of drug content localized to the needles

After drying, the amount of DOX located in each MN array was determined to be 0.81 ± 0.09 mg for MN-NP-1, 1.08 ± 0.11 for MN-NP-2, 0.82 ± 0.04 mg for MN-NP-3, 1.04 ± 0.08 mg for MN-NP-4 and 0.83 ± 0.05 mg for MN-NP-5. Accordingly, these drug amounts reflected the dosage of DOX in one MN array in the subsequent studies.

### 3.8 Investigation of the effect of MN formulation on the size and PDI of SLNs

One of the critical parameters in the formulation of NPs into dissolving MNs is the ability of the formulations to maintain the NP characteristics, specifically their sizes, PDIs and zeta potentials. Table 4 exhibits the NP properties in the MN formulations. These findings suggest that the incorporation NPs into MN arrays did not significantly (*p* > .05) affect the size, PDI and zeta potential of DOX-loaded NPs.

**Table 4:** Particle size, PDI and zeta potential of different formulations of DOX-loaded NPs in MN formulations (means ± SD, *n* = 3).

Formulations	Particle size (nm)	PDI	Zeta potential (mV)
NP-1	251.3 ± 11.3	0.223 ± 0.017	−5.6 ± 0.56
NP-2	211.6 ± 10.2	0.187 ± 0.013	−6.5 ± 0.98
NP-3	232.1 ± 19.3	0.205 ± 0.017	19.9 ± 1.87
NP-4	247.4 ± 12.7	0.214 ± 0.002	20.6 ± 2.02
NP-5	251.8 ± 9.1	0.221 ± 0.014	27.3 ± 1.72

### 3.9 *Ex vivo* dermatokinetic studies and antibiofilm activity in *ex vivo* model of biofilm on porcine skin

**Fig. 5:**
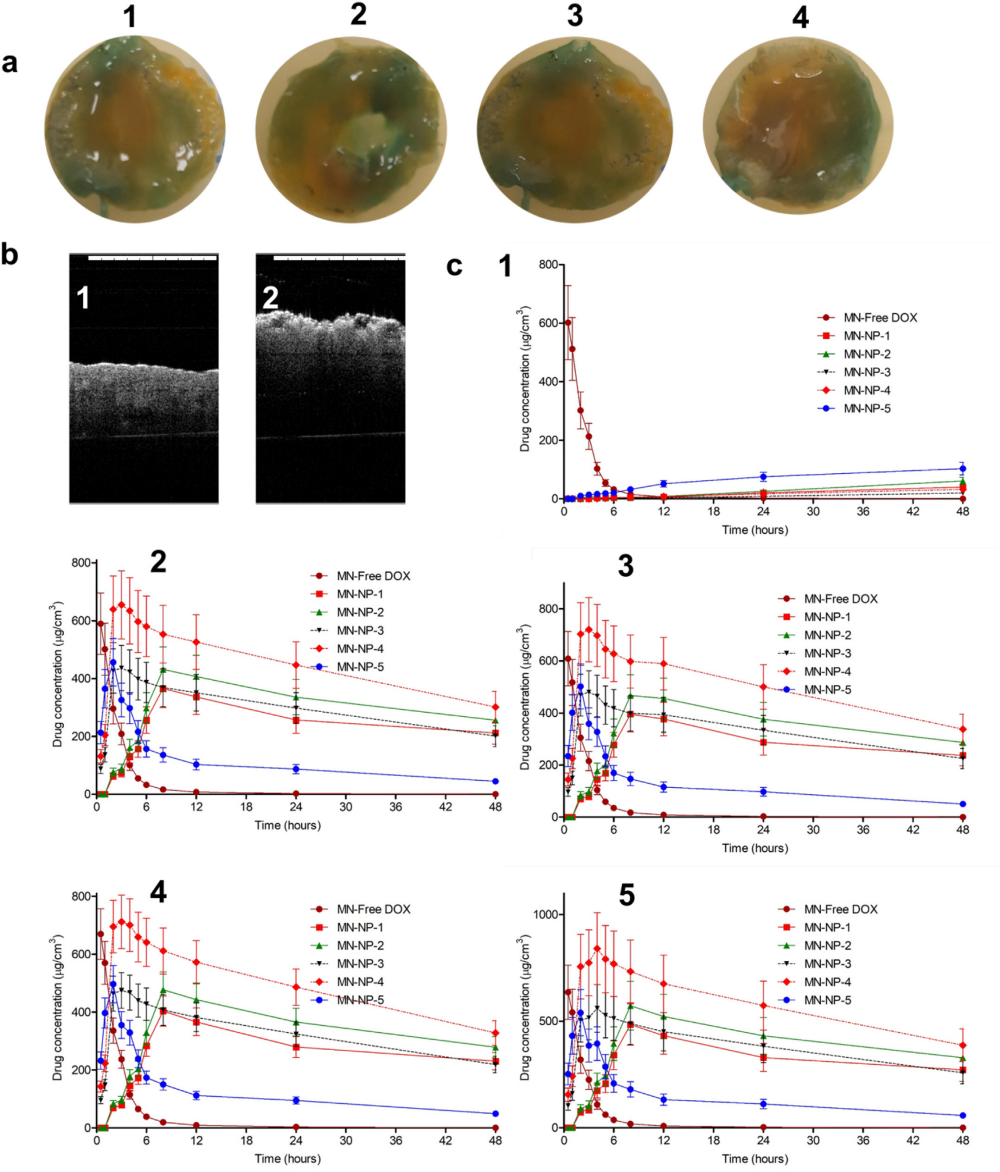
Representative images of *ex vivo* model of biofilm on full-thickness porcine skin (a) of SA1 (1), (SA2) (2), (PA1) (3) and (PA2) (4). OCT images of normal skin and *ex vvo* model of biofilm on porcine skin (b). The *ex vivo* DOX concentrations and time profiles (c) in non-infected full-thickness neonatal porcine (1), as well as *ex vivo* biofilm model in wound-1 (cut wound) formed by SA1 (2), SA2 (3), PA1 (4) and PA2 (5), following the application of MN containing free DOX and DOX-loaded NPs (means ± SD, *n* = 3).

**Fig. 6:**
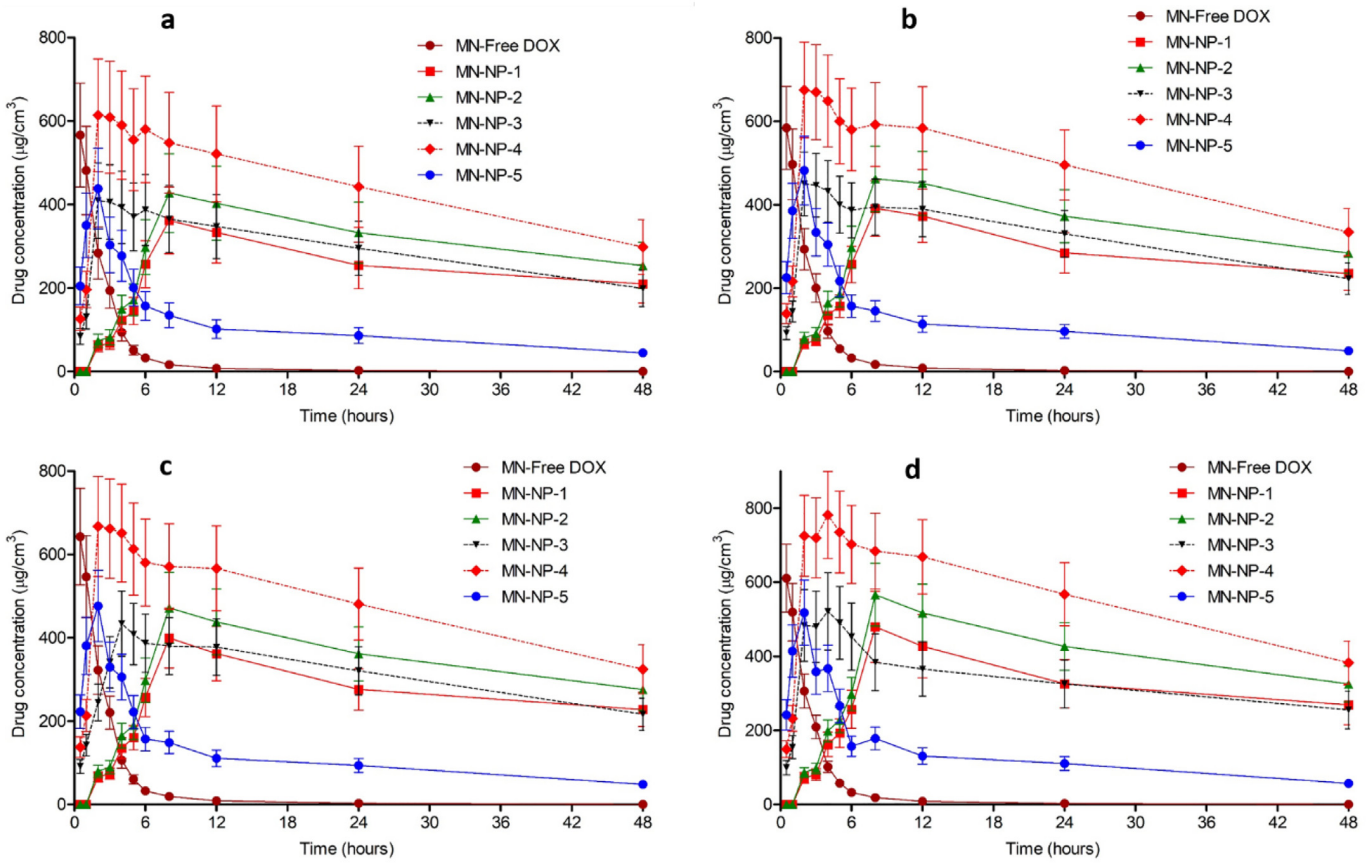
The *ex vivo* DOX concentrations and time profiles in *ex vivo* biofilm model in wound-2 (burn wound) formed by SA1 (a), SA2 (b), PA1 (c) and PA2 (d), following the application of MN containing free DOX and DOX-loaded NPs (means ± SD, *n* = 3). (means ± SD, *n* = 3).

To further understand the release kinetic profile of DOX from NPs in the dissolving MNs, a dermatokinetic study was performed in normal full-thickness porcine skin and *ex vivo* model of biofilm on the full-thickness porcine skin. Initially, *ex vivo* model of biofilm on full-thickness porcine skin was developed. These skin models are depicted in Fig. 5a. Dissolution studies of MN formulations containing drug-loaded NPs were investigated, with a view to predicting the time required for the needles to be dissolved in the skin during application. As shown in Fig. S1, dissolution of MNs while in place in the skin was reached by 20 min, with needles dissolved and a reduction in height obvious after 5 min. In addition, the models were also observed using OCT (Fig. 5b). The results showed that the normal neonatal porcine skin exhibited smooth surface and *ex vivo* model of biofilm wound exhibited uneven surface of the skin. OCT method has shown its effectiveness for cross-sectional observations of bacterial biofilm (Mohan et al., 2019). In order to ensure that the release of DOX was affected by the presence of bacterial biofilms, we quantified only the DOX released from NPs. For this purpose, the skin samples were homogenized at each time interval using water as a solvent. This method was also applied to NP dispersions to evaluate whether this method could disrupt the NPs. Our results showed that no DOX was detected in the supernatant of NP dispersions, showing that this method would only extract DOX released from NPs. This study was carried out in two types of biofilm model, namely cut wound biofilm (wound 1) and burn wound biofilm (wound 2). Four bacterial strains mentioned previously were used to form the biofilms. Figs. 5c and 6 illustrate the kinetic profiles of DOX in the non-infected skin and the *ex vivo* biofilm models, presented as the concentration *versus* the time of application, following the application of the MNs laden with five different DOX-loaded NPs formulations. We further analyzed the dermatokinetic profiles of DOX, namely C_max_, T_max_, T_1/2_, AUC and MRT, in these different formulations. The detail of the dermatokinetic profiles of DOX following the administration of the MN-free DOX and the MN-NPs laden with DOX are shown in Table S1-S5. In the non-infected skin, the release of DOX from the nanoparticles was negligible as compared to the free DOX, implying that the NPs could potentially avoid the non-specific release of DOX. Instead, in both biofilm models created from all bacterial strains, the release of DOX was significantly enhanced (*p* < .05) by the using of PLGA, PCL and chitosan in NP formulations. Specifically, despite non-significant difference (*p* > .05), the value of C_max_ of DOX from MN-NP-1, MN-NP-2 and MN-NP-5 were lower as compared to MN-NP-3 and MN-NP-4, respectively.

**Fig. 7:**
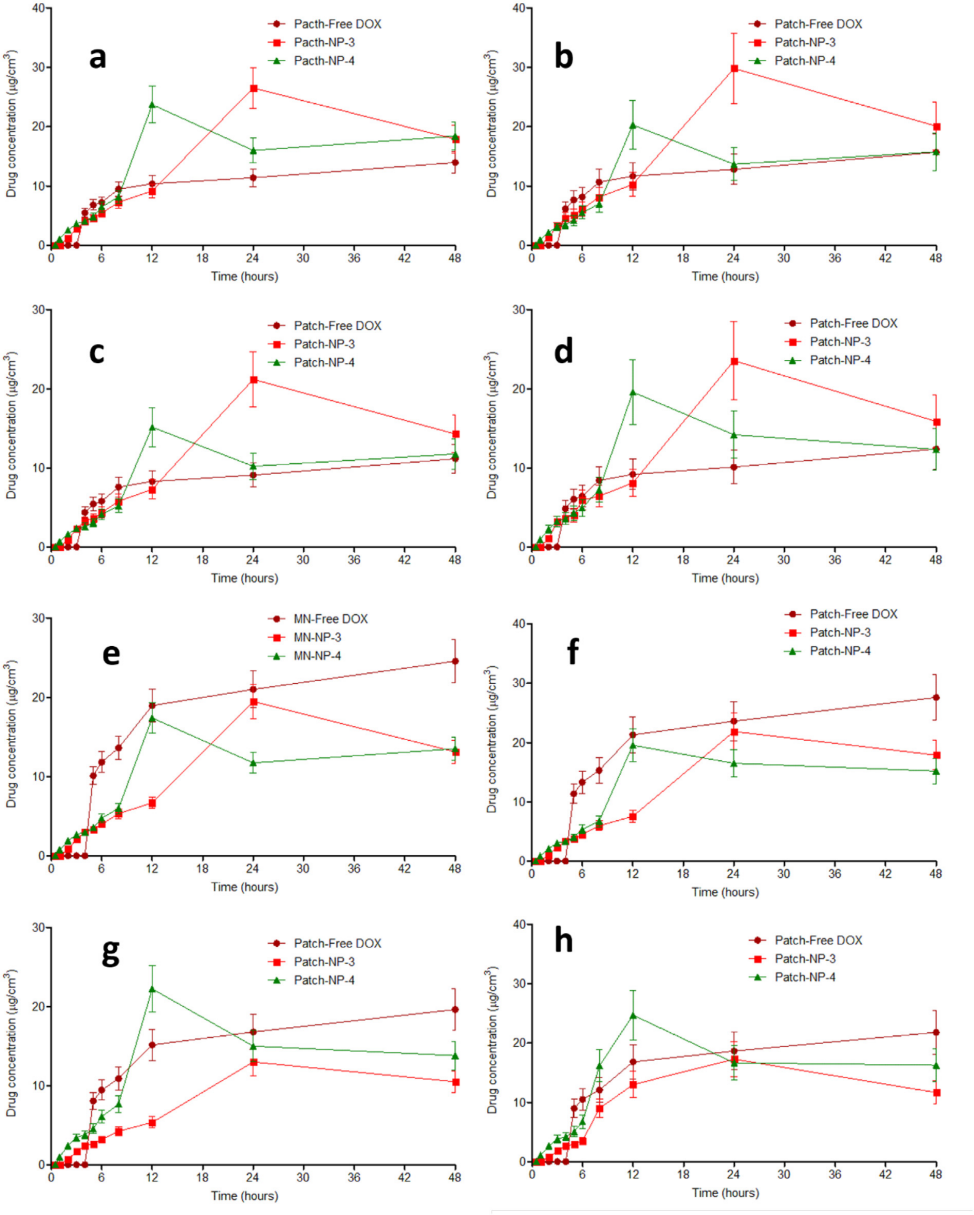
The *ex vivo* DOX concentrations and time profiles (b) in *ex vivo* biofilm model in wound-1 (cut wound) formed by SA1 (a), SA2 (b), PA1 (c) and PA2 (d), as well as *ex vivo* biofilm model in wound-2 (burn wound) formed by SA1 (e), SA2 (f), PA1 (g) and PA2 (h) following the application of needle-free patch containing free DOX and DOX-loaded NPs (means ± SD, *n* = 3). (means ± SD, *n* = 3).

**Fig. 8:**
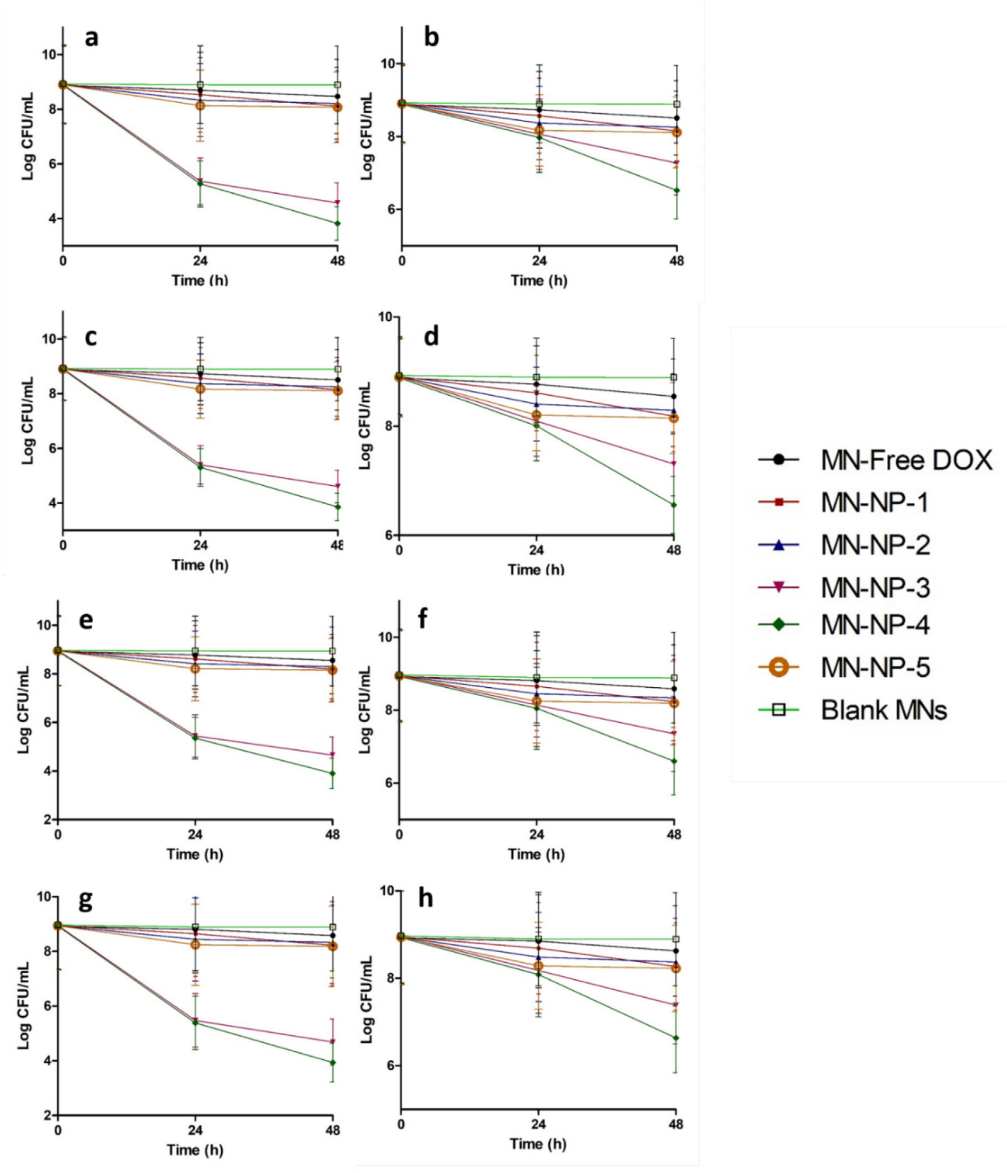
Bacterial viability (log CFU/mL) on in *ex vivo* biofilm model in wound-1 (cut wound) formed by SA1 (a), SA2 (b), PA1 (c) and PA2 (d), as well as biofilm *ex vivo* model in wound-2 (burn wound) formed by SA1 (e), SA2 (f), PA1 (g) and PA2 (h) following the application of blank MN and MN containing free DOX and DOX-loaded NPs (means ± SD, *n* = 3). At 24 h and 48 h post-application, *ex vivo* infected skin with biofilm were homogenized in sterile water and cultured onto TSA at 37 °C overnight.

The findings achieved here suggest that the encapsulation DOX in PLGA and PLC NPs coated chitosan could increase the concentration of DOX by two possible mechanisms. Initially, the outer layer of chitosan helped the NPs to be attached to the biofilm. Following this, the presence of lipase secreted by the bacterial strains degraded PLGA and PCL layers, leading to the release of DOX from NPs. With respect to T_max_, due to the reasons explained for the C_max_ results, the T_max_ values of DOX from MN-NP-3 and MN-NP-4 were found to be significantly lower (*p* < .05) compared to other NP formulations. In terms of the retention time in the skin, it was found that the MN containing free DOX showed MRT less than 3 h. Interestingly, the MRT values of DOX from MN-NPs were significantly greater (*p* < .05) than those in free DOX. This could be beneficial to reduce the application time of DOX in infected skin using this novel combinatorial approach, leading to patient acceptability of this device. In addition to this, we also performed the dermatokinetic studies for needle-free patches containing DOX and DOX-loaded NPs (Fig. 7). In this study, due to the higher DOX release from NP-3 and NP-4, only these NP formulations were selected as comparisons. The kinetic profiles of DOX from the patches are exhibited in Fig. 8. The results showed that, without MNs, NPs were not able to disrupt and penetrate the biofilms to release DOX, indicated by the significantly lower (*p* < .05) of DOX in all formulations delivered by the needle-free patches. Accordingly, the successful delivery of DOX into *ex vivo* biofilm model was a result of the combination of MN arrays and NPs.

**Fig. 9:**
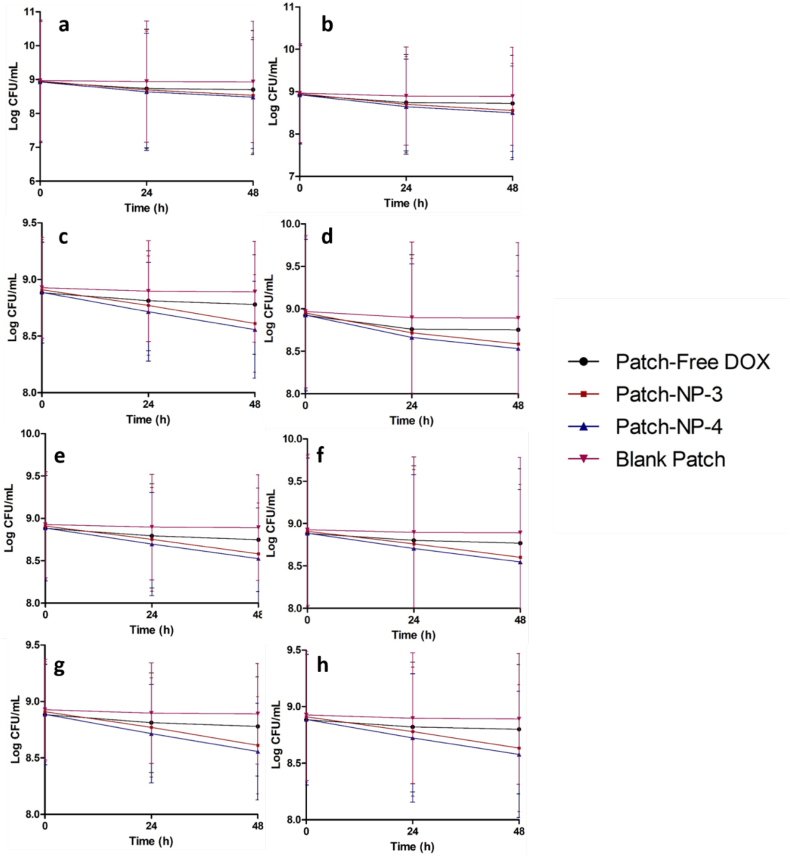
Bacterial viability (log CFU/mL) on in *ex vivo* biofilm model in wound-1 (cut wound) formed by SA1 (a), SA2 (b), PA1 (c) and PA2 (d), as well as biofilm *ex vivo* model in wound-2 (burn wound) formed by SA1 (e), SA2 (f), PA1 (g) and PA2 (h) following the application of blank patch and needle-free patch containing free DOX and DOX-loaded NPs (means ± SD, *n* = 3). At 24 h and 48 h post-application, *ex vivo* infected skin with biofilm were homogenized in sterile water and cultured onto TSA at 37 °C overnight.

Finally, to prove the efficacy of this delivery system, we evaluated the bacterial burden in an *ex vivo* biofilm models by viable cell counts. As depicted in Fig. 9, after the application of the blank MNs, the bacterial burden on biofilm wound models did not decrease following 48 h application time. On the other hand, the bacterial viabilities were reduced following 48 h of administration of MNs of DOX and DOX-loaded NPs (Fig. 8). In the case of the MN-free DOX, although the C_max_ was higher than MBC of DOX to all bacterial strains, the retention time of DOX after its administration was lower than the time required by DOX to kill the bacterial completely. Therefore, the bacterial burdens of all strains after the administration of MN-free DOX were reduced up to only ˜50%. With respect to MN-NP-1, MN-NP-2 and MN-NP-5, the killing rates were determined to be between 70% and 80%. Importantly, due to the better dermatokinetic profiles of these MNs compared to other formulations, more than 90% of bacterial burdens were killed after 48 h of the administration of MN-NP-3 and MN-NP-4. Specifically, ˜99.99% of SA1 and PA1 and ˜97% of SA2 and PA2 were killed in both biofilm models. In the control patches treatment, only around 60% of bacterial burden was reduced after 48 h treatment (Fig. 9). Our study is supported by Özcan et al. (2013), comparing the skin retention of betamethasone valerate from PLGA and chitosan NPs. It was found that the concentration of this drug in dermis following the application of PLGA NPs was around 4-times higher in comparison with chitosan NPs (Özcan et al. 2013). Therefore, these findings indicate that the combination of chitosan with PLGA and PCL in NPs could potentially improve not only dermatokinetic profiles of DOX, but also significantly enhance the efficacy of this system in *ex vivo* biofilm model in porcine skin by penetrating the biofilm in the infected skin and killing the bacteria.

Taken together, the findings presented here indicate that the decoration of PLGA and PCL NPs with chitosan could result in the specific delivery of DOX in the presence of bacterial cultures. Not only that, combined with dissolving MN arrays, but this unique approach also improved the penetration ability into bacterial biofilms in *ex vivo* biofilm models, indicated by the dermatokinetic profiles of DOX and the high bacterial burden reductions. The overriding advantage of the facilitated delivery system we have presented here, in comparison with needle-free patches, lies in the ability for site-specific delivery and long retention time in the infected skin which could potentially increase the effectiveness of antibacterial therapy of burns and chronic wounds. Following on from these promising findings, further studies are now required. Importantly, toxicity studies and *in vivo* efficacies study in a suitable infection animal models should also be carried out to completely exploit the potential applications of this system.

## 4 Conclusion

This study investigated the unique combination approach of bacterially sensitive NPs with dissolving MN arrays to enhance the antibiofilm properties of DOX. In NP preparations, PLGA and PCL were chosen as bacterially sensitive polymers coated with chitosan to improve the adhesion with bacterial biofilms. The NPs were spherical in shape with sizes around 200 nm. The formulation of DOX into PLGA and PCL NPs coated chitosan was able to enhance its antimicrobial and antibiofilm activity, compared to NPs prepared from only PLGA, PCL and chitosan. Furthermore, the release of DOX from the NPs was significantly affected by the presence of bacterial cultures, indicating the successful specific delivery of this system. Importantly, the incorporation of these NPs into MN arrays prepared from the combination of PVP and PVA resulted in MNs with sufficient mechanical properties and insertion abilities. Dermatokinetic studies indicated that the MNs was able improve the ability of NPs to penetrate bacterial biofilms in two *ex vivo* biofilm model in full-thickness porcine skin, namely biofilm of a burn wound and a cut wound, compared to a needle-free patch. Finally, the combination approach of PLGA and PCL NPs coated with chitosan with dissolving MNs showed proof of principle for the successful antibiofilm activity in *ex vivo* biofilm models, indicated by the reduction of bacterial bioburden >97%. However, further comprehensive studies are warranted, including toxicity studies and *in vivo* pharmacodynamic studies in a suitable infection animal model. In conclusion, before this delivery system can reach clinical practice and attain patient benefit, acceptability and usability investigations should also be performed to confirm maximum effect of the work.

## Author contributions

A.D.P and R.F.D conceived the research and design of all experiments. A.D.P, M. M and E.U performed the experiment. A.D.P wrote the manuscript. M. M and E.U helped with data analysis. A.D.P wrote the manuscript. R.F.D supervised the experiment and reviewed the manuscript thoroughly. All authors have given agreement

## Declaration of Competing Interest

The authors declare that they have no known competing financial interests or personal relationships that could have appeared to influence the work reported in this paper.

## Appendix A Supplementary data

Supplementary material Image 1
