# Supplementary material for: Bacterially sensitive nanoparticle-based dissolving microneedles of doxycycline for enhanced treatment of bacterial biofilm skin infection: A proof of concept study
Source: Int J Pharm X. 2020 Apr 14;2:100047. doi: 10.1016/j.ijpx.2020.100047 (PMC7168771; doi:10.1016/j.ijpx.2020.100047)
Supplement: Supplementary file 1 — Supplementary material [file mmc1.docx]

**Supporting information**

**Bacterially sensitive nanoparticle-based dissolving microneedles of doxycycline for enhanced treatment of bacterial biofilm skin infection: A proof of concept study**

Andi Dian Permana^1,2^, Maria Mir^1,3^, Emilia Utomo^1^, Ryan F. Donnelly^1*^

1. School of Pharmacy, Queen's University Belfast, Medical Biology Centre, 97 Lisburn Road, Belfast. BT9 7BL, UK
2. Department of Pharmaceutics, Faculty of Pharmacy, Hasanuddin University, Makassar, Indonesia
3. Department of Pharmacy, Faculty of Biological Sciences, Quaid-i-Azam University, Islamabad 45320, Pakistan

***Corresponding author:**

**Professor Ryan F. Donnelly**

**Chair in Pharmaceutical Technology**

**School of Pharmacy**

**Queens University Belfast**

**Medical Biology Centre**

**97 Lisburn Road**

**Belfast**

**BT9 7BL, Northern Ireland**

**United Kingdom**

**Tel: +44 (0) 28 90 972 251**

**Fax: +44 (0) 28 90 247 794**

**Email: r.donnelly@qub.ac.uk**

**
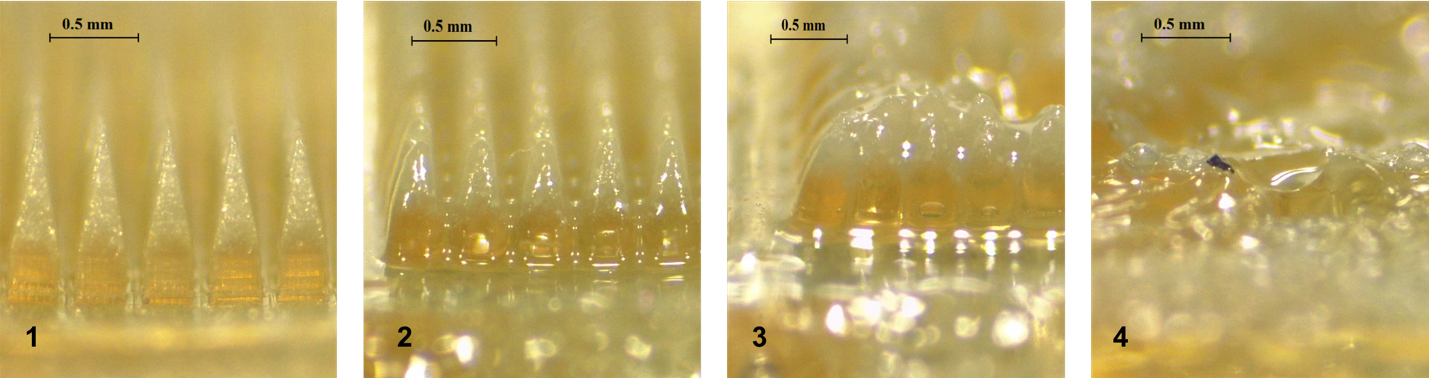
**

**Figure S1.** Illustrative of digital micrographs of the dissolution of MN formulations NP-1 at 0 (1,) 5 min (2), 10 min (3) and 20 min (4), following insertion into and removal from *ex vivo* model of biofilm on full-thickness porcine skin

**Table S1.** *Ex vivo* dermatokinetic parameters of DOX following the administration of the MN-free DOX and the MN-NPs laden with DOX in normal excised full thickness porcine skin (means ± SD, *n* = 3).

| Condition | Formulation | Cmax (μg/cm^3^) | Tmax (h) | T1/2 (h) | AUC (h.(μg/cm^3^) | MRT (h) |
| --- | --- | --- | --- | --- | --- | --- |
| Normal skin | MN-Free DOX | 601.45 ± 103.4 | 0.501 ± 0.02 | 1.36 ± 0.01 | 1517.36 ± 273.23 | 2.32 ± 0.25 |
|  | MN-NP-1 | 21.73 ± 5.33 | 67.76 ± 7.54 | 50.5 ± 6.87 | 635.31 ± 76.45 | 136.06 ± 21.43 |
|  | MN-NP-2 | 26.72 ± 4.32 | 59.98 ± 9.65 | 44.64 ± 7.16 | 819.58 ± 100.32 | 120.35 ± 22.71 |
|  | MN-NP-3 | 7.63 ± 0.87 | 39.67 ± 4.32 | 28.72 ± 3.61 | 280.87 ± 33.17 | 79.41 ± 8.54 |
|  | MN-NP-4 | 16.32 ± 2.12 | 52.33 ±8.11 | 38.13 ± 4.09 | 543.11 ± 62.32 | 104.3 ± 28.65 |
|  | MN-NP-5 | 77.98 ± 12.31 | 40.38 ± 8.21 | 29.67 ± 4.12 | 2851.21 ± 432.12 | 80.32 ± 13.76 |

**Table S2.** *Ex vivo* dermatokinetic parameters of DOX following the administration of the MN-free DOX and the MN-NPs laden with DOX in *ex vivo* model of biofilm on full-thickness porcine skin (wound 1) of SA1 and SA2 (means ± SD, *n* = 3).

| Condition | Formulation | Cmax (μg/cm^3^) | Tmax (h) | T1/2 (h) | AUC (h.(μg/cm^3^) | MRT (h) |
| --- | --- | --- | --- | --- | --- | --- |
| Wound 1 (SA1) | MN-Free DOX | 589.87 ± 98.43 | 0.499 ± 0.03 | 1.35 ± 0.02 | 1499.12 ± 203.43 | 2.16 ± 0.32 |
|  | MN-NP-1 | 328.32 ± 61.23 | 17.51 ± 2.18 | 26.48 ± 4.87 | 12395.43 ± 2871.43 | 47.62 ± 5.43 |
|  | MN-NP-2 | 407.43 ± 81.32 | 18.29 ± 2.65 | 23.39 ± 4.18 | 15341.12 ± 2133.87 | 44.75 ± 6.19 |
|  | MN-NP-3 | 410.61 ± 76.11 | 5.23 ± 0.98 | 36.55 ± 5.22 | 14027.32 ± 2032.41 | 54.15 ± 7.21 |
|  | MN-NP-4 | 615.65 ± 98.09 | 5.65 ± 1.21 | 38.12 ± 4.12 | 21040.87 ± 3872.02 | 56.43 ± 8.12 |
|  | MN-NP-5 | 400.32 ± 76.19 | 1.73 ± 0.27 | 3.22 ± 0.54 | 2706.32 ± 398.18 | 5.48 ± 1.32 |
| Wound 1 (SA2) | MN-FREE DOX | 608.82 ± 98.32 | 0.498 ± 0.04 | 1.31 ± 0.01 | 1543.5 ± 201.32 | 2.16 ± 0.41 |
|  | MN-NP-1 | 364.61 ± 32.31 | 17.86 ± 1.21 | 26.38 ± 3.02 | 13790.58 ± 2653.11 | 47.82 ± 6.32 |
|  | MN-NP-2 | 452.95 ± 81.09 | 18.67 ± 1.32 | 22.83 ± 2.09 | 17079.48 ± 2091.87 | 44.52 ± 5.18 |
|  | MN-NP-3 | 448.59 ± 54.22 | 5.24 ± 0.67 | 38.63 ± 4.93 | 15565.06 ± 1921.21 | 57.12 ± 7.98 |
|  | MN-NP-4 | 672.89 ± 12.98 | 5.24 ± 0.59 | 37.53 ± 3.88 | 23347.6 ± 2343.98 | 57.12 ± 8.09 |
|  | MN-NP-5 | 440.66 ± 76.43 | 1.73 ± 0.11 | 3.17 ± 0.41 | 2946.18 ± 432.19 | 5.41 ± 0.49 |

**Table S3.** *Ex vivo* dermatokinetic parameters of DOX following the administration of the MN-free DOX and the MN-NPs laden with DOX in *ex vivo* model of biofilm on full-thickness porcine skin (wound 1) of PA1 and PA2 (means ± SD, *n* = 3).

| Condition | Formulation | Cmax (μg/cm^3^) | Tmax (h) | T1/2 (h) | AUC (h.(μg/cm^3^) | MRT (h) |
| --- | --- | --- | --- | --- | --- | --- |
| Wound 1 (PA1) | MN-FREE DOX | 669.7 ± 90.09 | 0.481 ± 0.03 | 1.38 ± 0.02 | 1697.84 ± 302.87 | 2.16 ± 0.41 |
|  | MN-NP-1 | 359 ± 54.43 | 17.34 ± 1.17 | 26.48 ± 2.77 | 13515.09 ± 2100.32 | 47.48 ± 7.65 |
|  | MN-NP-2 | 444.89 ± 76.12 | 18.12 ± 1.98 | 23.58 ± 2.13 | 16721.91 ± 2102.87 | 44.79 ± 8.01 |
|  | MN-NP-3 | 451.08 ± 61.43 | 5.27 ± 0.67 | 35.66 ± 4.09 | 15308.49 ± 2187.44 | 52.87 ± 10.31 |
|  | MN-NP-4 | 676.62 ± 87.21 | 4.98 ± 0.71 | 31.61 ± 3.91 | 22962.74 ± 3087.19 | 51.98 ± 9.76 |
|  | MN-NP-5 | 435.49 ± 32.41 | 1.74 ± 0.21 | 3.29 ± 0.43 | 2986.2 ± 198.11 | 5.57 ± 0.77 |
| Wound 1 (PA2) | MN-FREE DOX | 636.21 ± 100.32 | 0.49 ± 0.05 | 1.35 ± 0.02 | 1612.95 ± 201.39 | 2.16 ± 0.41 |
|  | MN-NP-1 | 425.99 ± 87.19 | 17.28 ± 1.98 | 26.01 ± 3.19 | 16002.81 ± 1862.18 | 48.86 ± 7.18 |
|  | MN-NP-2 | 527.61 ± 101.19 | 18.06 ± 1.91 | 23.22 ± 3.04 | 19795.58 ± 2187.51 | 44.32 ± 8.01 |
|  | MN-NP-3 | 530.57 ± 87.33 | 5.75 ± 0.65 | 35.32 ± 4.12 | 18084.19 ± 2115.32 | 52.58 ± 10.53 |
|  | MN-NP-4 | 795.86 ± 121.09 | 5.13 ± 0.43 | 31.98 ± 3.79 | 27126.29 ± 1198.43 | 50.87 ± 9.41 |
|  | MN-NP-5 | 473.4 ± 54.32 | 1.79 ± 0.19 | 3.9 ± 0.43 | 3663.07 ± 543.13 | 6.4 ± 1.09 |

**Table S4.** *Ex vivo* dermatokinetic parameters of DOX following the administration of the MN-free DOX and the MN-NPs laden with DOX in *ex vivo* model of biofilm on full-thickness porcine skin (wound 2) of SA1 and SA2 (means ± SD, *n* = 3).

| Condition | Formulation | Cmax (μg/cm^3^) | Tmax (h) | T1/2 (h) | AUC (h.(μg/cm^3^) | MRT (h) |
| --- | --- | --- | --- | --- | --- | --- |
| Wound 2 (SA1) | MN-FREE DOX | 566.24 ± 100.09 | 0.479 ± 0.03 | 1.29 ± 0.21 | 1417.05 ± 201.76 | 2.12 ± 0.41 |
|  | MN-NP-1 | 325.2 ± 54.79 | 17.77 ± 1.19 | 25.61 ± 3.14 | 12259.93 ± 2812.31 | 46.82 ± 8.13 |
|  | MN-NP-2 | 403.59 ± 65.33 | 18.57 ± 2.09 | 22.25 ± 2.65 | 15177.61 ± 2632.19 | 43.79 ± 7.42 |
|  | MN-NP-3 | 394.7 ± 50.98 | 5.44 ± 0.71 | 39.27 ± 4.14 | 13790.84 ± 3764.16 | 58.14 ± 10.11 |
|  | MN-NP-4 | 592.06 ± 43.11 | 5.11 ± 0.49 | 37.73 ± 3.77 | 20686.25 ± 4012.32 | 53.98 ± 7.18 |
|  | MN-NP-5 | 378.4 ± 49.29 | 1.69 ± 0.02 | 3.43 ± 0.44 | 2639.73 ± 503.42 | 5.72 ± 10.32 |
| Wound 2 (SA2) | MN-FREE DOX | 584.38 ± 77.32 | 0.449 ± 0.07 | 1.33 ± 0.29 | 1464.09 ± 214.18 | 2.11 ± 0.42 |
|  | MN-NP-1 | 359.09 ± 65.31 | 18.39 ± 1.99 | 25.09 ± 3.03 | 13594.68 ± 2113.48 | 46.79 ± 5.43 |
|  | MN-NP-2 | 446.87 ± 35.44 | 19.2 ± 2.01 | 20.87 ± 2.98 | 16849.53 ± 3014.35 | 43.1 ± 7.65 |
|  | MN-NP-3 | 425.34 ± 98.33 | 5.38 ± 0.61 | 43.14 ± 5.31 | 15213.42 ± 2198.32 | 63.62 ± 8.43 |
|  | MN-NP-4 | 638.01 ± 100.65 | 5.09 ± 0.44 | 41.32 ± 6.32 | 22820.12 ± 4137.43 | 61.09 ± 10.32 |
|  | MN-NP-5 | 417.5 ± 65.87 | 1.69 ± 0.19 | 3.25 ± 0.43 | 2812.11 ± 319.73 | 5.48 ± 1.09 |

**Table S5.** *Ex vivo* dermatokinetic parameters of DOX following the administration of the MN-free DOX and the MN-NPs laden with DOX in *ex vivo* model of biofilm on full-thickness porcine skin (wound 2) of PA1 and PA2 (means ± SD, *n* = 3).

| Condition | Formulation | Cmax (μg/cm^3^) | Tmax (h) | T1/2 (h) | AUC (h.(μg/cm^3^) | MRT (h) |
| --- | --- | --- | --- | --- | --- | --- |
| Wound 2 (PA1) | MN-FREE DOX | 642.77 ± 112.31 | 0.5 ± 0.04 | 1.33 ± 0.02 | 1607.56 ± 203.46 | 2.09 ± 0.39 |
|  | MN-NP-1 | 352.78 ± 98.78 | 17.93 ± 1.81 | 25.33 ± 2.04 | 13307.26 ± 2013.41 | 46.62 ± 6.28 |
|  | MN-NP-2 | 438.1 ± 76.89 | 18.72 ± 2.04 | 21.79 ± 1.98 | 16478.87 ± 2098.19 | 43.47 ± 8.09 |
|  | MN-NP-3 | 406.36 ± 81.98 | 7.41 ± 0.59 | 40.94 ± 5.18 | 14680.75 ± 2032.76 | 61.22 ± 6.49 |
|  | MN-NP-4 | 632.86 ± 109.34 | 5.31 ± 0.44 | 40.72 ± 6.11 | 22289.18 ± 4174.63 | 60.14 ± 10.02 |
|  | MN-NP-5 | 412.7 ± 43.98 | 1.7 ± 0.21 | 3.35 ± 0.52 | 2836.47 ± 418.41 | 5.61 ± 1.43 |
| Wound 2 (PA2) | MN-FREE DOX | 610.66 ± 87.18 | 0.5 ± 0.04 | 1.29 ± 0.21 | 1528.66 ± 298.36 | 2.21 ± 0.44 |
|  | MN-NP-1 | 414.21 ± 67.98 | 18.39 ± 2.13 | 24.21 ± 4.12 | 15641.07 ± 2218.39 | 45.78 ± 10.87 |
|  | MN-NP-2 | 515.25 ± 65.43 | 19.19 ± 1.76 | 20.04 ± 4.01 | 19383.52 ± 2198.74 | 42.34 ± 8.43 |
|  | MN-NP-3 | 467.04 ± 76.31 | 5.15 ± 0.05 | 37.25 ± 4.32 | 16023.38 ± 3015.44 | 65.12 ± 8.65 |
|  | MN-NP-4 | 745.71 ± 98.67 | 4.93 ± 0.05 | 32.31 ± 3.18 | 26335.64 ± 4021.32 | 59.54 ± 8.75 |
|  | MN-NP-5 | 449.51 ± 67.87 | 1.73 ± 0.02 | 3.71 ± 4.08 | 3332.29 ± 523.51 | 6.12 ± 0.98 |
